# Role of SARS-CoV-2-specific memory B cells promoting immune protection after booster vaccination in solid organ transplantation

**DOI:** 10.3389/fimmu.2024.1463769

**Published:** 2024-10-08

**Authors:** Laura Donadeu, Susana Gomez-Olles, Franc Casanova, Alba Torija, Manuel Lopez-Meseguer, Meritxell Boada-Pérez, Delphine Kervella, Elena Crespo, Claudia Carrera-Muñoz, Isabel Campos-Varela, Lluís Castells, Maria F. Cortese, Juliana Esperalba, Candela Fernández-Naval, Jesús Quintero, Marina Muñoz, Fernando Agüero, José Gonzalez-Costello, Laura Lladó, Alexandre Favà, Laura Cañas, María del Mar de la Hoz-Caballero, Maria Meneghini, Irina B. Torres, Mariona Juvé, FMJ Hafkamp, Marta Vila, Alba G. Robles, Maria José Buzón, Nestor Toapanta, José Miguel Zúñiga, Víctor Monforte, Berta Saez-Giménez, Oscar Len, Ibai Los Arcos, Enric Miret, Gema Ariceta, Emma Pardo, Xavier Martínez, Francesc Moreso, Oriol Bestard

**Affiliations:** ^1^ Laboratory of Nephrology and Transplantation, Vall d’Hebron Institut de Recerca (VHIR), Universitat Autònoma de Barcelona, Barcelona, Spain; ^2^ Vall d’Hebron for Solid Organ Transplantation Research Group, Vall d’Hebron Institut de Recerca (VHIR), Universitat Autònoma de Barcelona, Barcelona, Spain; ^3^ Laboratory of Pneumology, Vall d’Hebron Institut de Recerca (VHIR), Universitat Autònoma de Barcelona, Barcelona, Spain; ^4^ Ciber Enfermedades Respiratorias (CIBERES), Madrid, Spain; ^5^ Lung Transplant Unit, Pneumology Department, Vall d’Hebron Hospital Universitari, Universitat Autònoma de Barcelona, Barcelona, Spain; ^6^ Kidney Transplant Unit, Nephrology Department, Vall d’Hebron Hospital Universitari, Universitat Autònoma de Barcelona, Barcelona, Spain; ^7^ Liver Unit, Vall d'Hebron Hospital Universitari, Universitat Autònoma de Barcelona, Barcelona, Spain; ^8^ Centro de Investigación Biomédica en Red de Enfermedades Hepáticas y Digestivas (CIBERehd), Instituto de Salud Carlos III, Madrid, Spain; ^9^ Microbiology Department, Vall d’Hebron Hospital Universitari, Universitat Autònoma de Barcelona, Barcelona, Spain; ^10^ Pediatric Hepatology and Liver Transplant Department, Vall d’Hebron Hospital Universitari, Universitat Autònoma de Barcelona, Barcelona, Spain; ^11^ Department of Pediatric Nephrology, Vall d’Hebron Hospital Universitari, Universitat Autònoma de Barcelona, Barcelona, Spain; ^12^ Department of Preventive Medicine and Epidemiology, Bellvitge University Hospital, Barcelona, Spain; ^13^ Advanced Heart Failure and Heart Transplant Unit, Department of Cardiology, Hospital Universitari de Bellvitge, BIOHEART-Cardiovascular Diseases Research Group, Bellvitge Biomedical Research Institute (IDIBELL), Universitat de Barcelona, Ciber Cardiovascular (CIBERCV), Barcelona, Spain; ^14^ Liver Transplant Unit, Bellvitge University Hospital, Barcelona, Spain; ^15^ Kidney Transplant Unit, Bellvitge University Hospital, Barcelona, Spain; ^16^ Kidney Transplant Unit, Nephrology department, Germans Trias i Pujol Hospital, Badalona, Spain; ^17^ Equipo de Atención Primaria Sant Rafael, Servei d'Atenció Primària (SAP) Muntanya, Gerència Territorial de Barcelona Ciutat, Institut Català de la Salut, Barcelona, Spain; ^18^ Infectious Diseases Department, Vall d’Hebron Institut de Recerca (VHIR), Universitat Autònoma de Barcelona, Barcelona, Spain; ^19^ Department of Infectious Diseases, Vall d’Hebron Hospital Universitari, Universitat Autònoma de Barcelona, Barcelona, Spain; ^20^ Urology Department, Vall d’Hebron Hospital Universitari, Vall d’Hebron Institut de Recerca (VHIR), Universitat Autònoma de Barcelona, Barcelona, Spain; ^21^ Department of Preventive Medicine and Epidemiology, Vall d’Hebron Hospital Universitari, Vall d’Hebron Institut de Recerca (VHIR), Universitat Autònoma de Barcelona, Barcelona, Spain

**Keywords:** SARS-CoV-2, booster vaccination, solid organ transplantation, adaptive immunity, neutralizing antibodies

## Abstract

**Introduction:**

Solid organ transplant (SOT) recipients display weak seroconversion and neutralizing antibody (NAb) responses after severe acute respiratory syndrome coronavirus 2 (SARS-CoV-2) vaccination and remain at risk of severe coronavirus disease 2019 (COVID-19). While B-cell memory is the hallmark of serological immunity, its role in driving successful vaccine responses and providing immune protection in SOT patients remains unclear.

**Methods:**

We investigated the function and interplay of SARS-CoV-2-specific memory B cells (mBc), different cytokineproducing T cells, and cross-reactive NAb in driving seroconversion and protection against COVID-19 in two cohorts. First, we studied a large cohort of 148 SOT recipients and 32 immunocompetent individuals who underwent several vaccinations. Subsequently, we assessed 25 SOT patients participating in a randomized controlled trial to compare two different immunosuppressive strategies for allowing successful seroconversion and memory-cell responses after booster vaccination.

**Results:**

We corroborate previous findings that B- and T-cell memory responses are weaker and more delayed in SOT patients than in immunocompetent (IC) individuals; however, within the SOT cohort, we found that these responses are relatively stronger and more robust in patients not receiving mycophenolate mofetil (MMF)-based therapies. Anti- spike IgG titers strongly correlated with RBD-specific IgG-producing mBc, with both displaying broad viral cross reactivity. Prebooster SARS-CoV-2-specific mBc and IL-2- producing T cells accurately predicted Nab seroconversion (AUC, 0.828) and protection against severe COVID-19. While switching unresponsive SOT patients from calcineurin inhibitors (CNI)/MMF to a low-exposure CNI/mTOR-i regimen favored wider SARS-CoV-2-specific immune responses after a fourth booster vaccination, preformed RBD-specific mBc predicted NAb seroconversion.

**Discussion:**

Our study adds new insights into the pathobiology of immune memory and highlights the pivotal role of SARS-CoV-2-specific mBc in promoting immune protection inSOT patients.

## 1 Introduction

With the extraordinarily fast development and implementation of active immunization with new vaccines against severe acute respiratory syndrome coronavirus 2 (SARS-CoV-2), a profound decline in coronavirus disease 2019 (COVID-19), especially the more severe clinical forms, has been achieved worldwide (1–4). Despite the global protective effect of SARS-CoV-2 vaccination (5, 6), high-risk patient populations, such as solid organ transplantation (SOT) recipients, remain significantly exposed to recurrent and more severe SARS-CoV-2 breakthrough infections (BTI) (7–12). This is due to markedly impaired and short- lived humoral and cellular immune responses following booster vaccination compared to immunocompetent (IC) individuals (13–18).

While serological immunity, characterized by neutralizing antibodies (NAb) specific to SARS-CoV- 2, is considered the hallmark of immune protection from symptomatic COVID-19 (19–21), different cellular immune compartments, such as SARS-CoV-2-specific cytotoxic T cells, play a complementary role by providing protection through the rapid expansion and elimination of virus-infected cells (22).

Importantly, a key feature of humoral immunity is the generation of antigen-specific B cells that harbor highly specific and affine B-cell receptors, which are the source of the outstanding adaptability of these immune cells. Subsequently, after clonal selection and somatic hypermutation, these B cells eventually give rise to plasma cells that produce high-affinity antibodies (23). Indeed, recent studies in mice and healthy individuals have highlighted the role of SARS-CoV-2-specific memory B cells (mBc) in driving successful seroconversion with NAb and protection against COVID-19 BTI after booster vaccination (24–28). However, we and others have shown the significantly poorer capacity of SOT to develop long-lasting T and especially B-cell memory responses after primary infection (29–32), as well as after several booster mRNA-based vaccinations (33–35).

To avoid allograft rejection, SOT patients receive different long-lasting immunosuppressive treatments, including calcineurin inhibitors (CNI; cyclosporine or tacrolimus), mammalian target of rapamycin (mTOR) inhibitors (rapamycin, everolimus), antimetabolites such as inosine monophosphate dehydrogenase Inhibitors (mycophenolate acids, mycophenolate mofetil (MMF)), and corticosteroids. While all of them abrogate alloreactive immune responses at different cellular levels (36), the lack of antigen-specific targeting function similarly inhibits protective antiviral immune responses. Interestingly, while high CNI trough levels and the use of MMF seem to not favor the development of antiviral humoral and cellular responses after infection or vaccination (37, 38), the use of mTOR inhibitor (mTOR-i) has been shown to boost antiviral cellular immune responses after viral infection or vaccination in different settings (13, 39, 40). Therefore, assessing the impact of current immunosuppressive regimens on SARS-CoV-2-specific humoral and cellular immune responses after repeated vaccinations, identifying robust independent immune correlates of successful immunization, and investigating safe immunosuppressive strategies favoring antiviral protective immunity are highly warranted for this high-risk patient population.

Herein, we aimed to thoroughly characterize the role and cross-reactivity of SARS-CoV-2-specific humoral and memory-cell responses in a large cohort of SOT patients receiving multiple booster vaccinations. We sought to decipher their role in predicting successful immunization and protection from severe forms of COVID-19. Additionally, we designed a mechanistic randomized controlled trial (RCT) to assess whether switching from CNI/MMF to a low-exposure CNI/mTOR-i immunosuppressive regimen would favor protective humoral and cellular immunity after a fourth booster vaccination among SOT unresponsive to three previous vaccine doses.

## 2 Materials and methods

### 2.1 Patients of the study and clinical definitions

**Figure 1:**
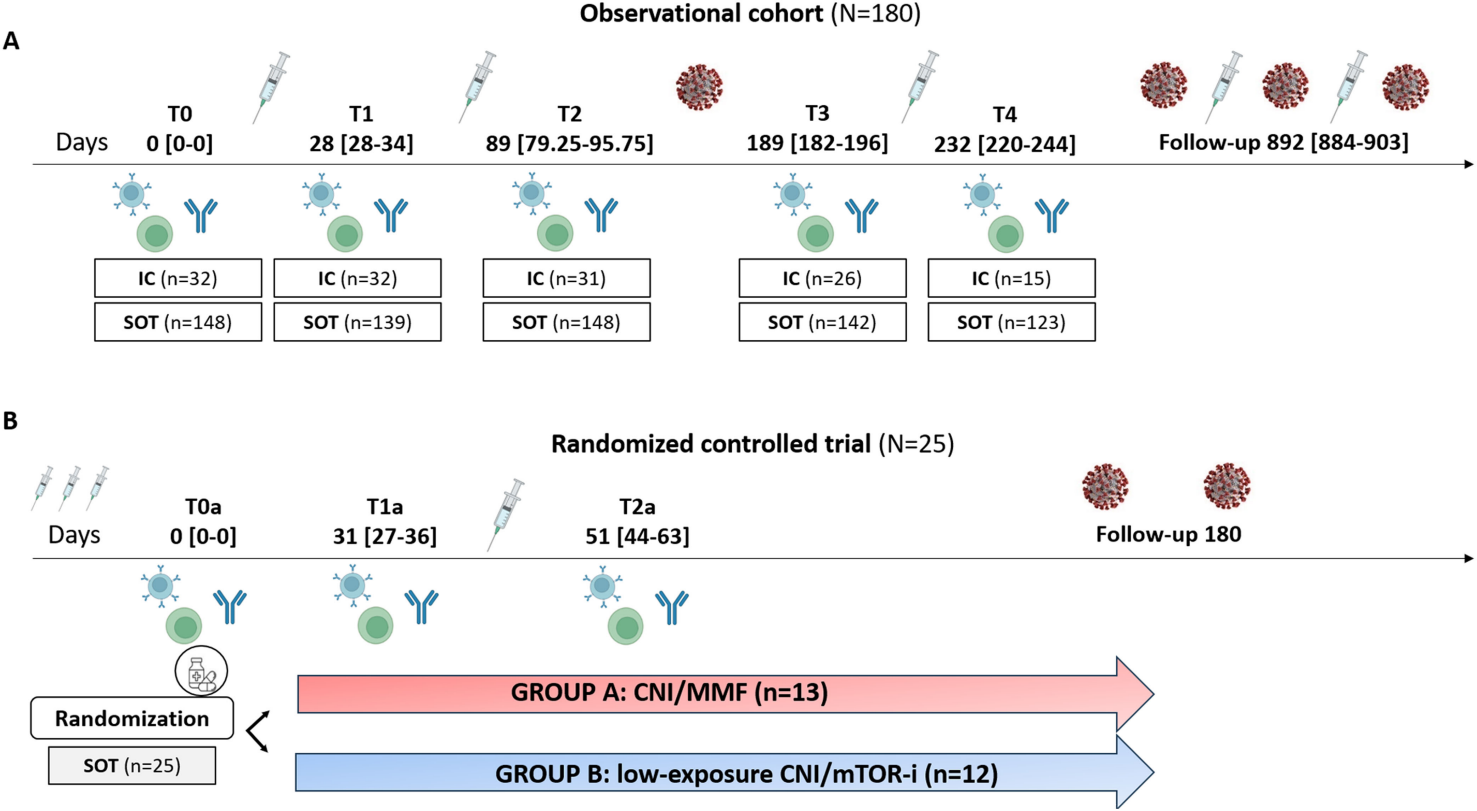
Flowchart of the study. **(A)** Observational cohort. **(B)** Randomized controlled trial. IC, immunocompetent; SOT, solid organ transplant; CNI, calcineurin inhibitors; MMF, micophenolate mofetil; mTOR-i, mTOR inhibitors.

The study included two distinct cohorts of SOT recipients; (i) a first large, multicenter, prospective observational cohort of SOT and IC patients who underwent three consecutive SARS-CoV-2 booster vaccinations and were monitored for distinct serological and cellular immune responses specific to the virus, and (ii) a second cohort of SOT group who, despite receiving three previous vaccine doses, did not have neutralizing SARS-CoV-2-specific IgG antibodies (NAb). These patients participated in an interventional RCT to assess the achievement of NAb and cellular immune memory after a fourth vaccine dose, comparing a standard- of-care immunosuppressive regimen based on CNI/MMF (MMF/mycophenolate sodium (MPS)) with a regimen switched to a CNI/mTOR -i-based immunosuppressive therapy (**Figure 1**).

#### 2.1.1 Observational cohort

This large multicenter patient cohort consisted of 836 serial peripheral blood samples from 148 adult and adolescent (15–18 years old) SOT patients from three different transplant centers (Vall d’Hebron, Bellvitge, and Germans Trias I Pujol University Hospitals) as well as 32 IC individuals who received early vaccination according to national health policies. These individuals were used as controls and were all prospectively evaluated at five different time points, both prior and after three consecutive mRNA-based vaccine doses against SARS-CoV-2 (**Figure 1**). SOT patients received different immunosuppressive regimens: 85 were on CNI (tacrolimus or cyclosporine) plus MMF or MPS, eight were on MMF monotherapy, 33 were only on CNI, and 20 received a CNI plus a mTOR inhibitor (**Table 1**). Main clinical, demographic, and immunological patient characteristics were recorded. The study was approved by each ethical review board (PR115/20), and patient recruitment was done after obtaining a signed informed consent.

**Table 1:** Main demographic and clinical characteristics of the discovery population.

Patients of the observational study (*N* = 180)	SOT (*N* = 148)	IC (*N* = 32)	*p*-value
Age (years, median [IQR])	58 [38.25–69]	73 [50–74]	0.023
Sex (female; *n* (%))	57 (38.51)	18 (56.25)	0.065
Time after transplant (months, median [IQR])	69.63 [24.08–148.43]	NA	–
Organ (*n* (%))			
- Kidney	54 (36.49)	NA	–
- Liver	42 (28.38)	NA	–
- Lung	28 (18.92)	NA	–
- Heart	24 (16.22)	NA	–
Induction treatment (*n* (%))			
- No	72 (48.65)	NA	–
- Anti-thymocyte globulins	29 (19.59)	NA	–
- Anti-CD25	43 (29.05)	NA	–
- Others	4 (2.70)	NA	–
Maintenance immunosuppression (*n* (%))			
- CNI (TAC or CsA) + MMF/MPS	85 (57.43)	NA	–
- MMF/MPS	8 (5.41)	NA	–
- CNI (TAC or CsA)	33 (22.30)	NA	–
- CNI (TAC or CsA) + mTOR-i	20 (14.86)	NA	–
- Steroids	94 (63.51)	NA	–
Main comorbidities (*n* (%))			
- Diabetes	34 (23.10)	4 (10.50)	0.183
- Arterial hypertension	61 (41.20)	10 (31.20)	0.296
- Obesity	16 (10.81)	3 (9.40)	0.747
- Active neoplasm	2 (1.35)	0 (0)	0.472
mRNA vaccine (%)			
- mRNA-1273 (Moderna)	134 (90.54)	12 (37.50)	< 0.001
- BNT162b2 (Pfizer-BioNTech)	14 (9.46)	20 (62.50)	
Immune monitoring times (days; median [IQR])			
- T0	0 [0–0]	0 [0–0]	1.000
- T1	28 [28–34]	21 [21–23]	< 0.001
- T2	92 [83–96]	77 [77–79]	< 0.001
- T3	191 [186–196]	168 [167.50–170]	< 0.001
- T4	232 [222–244]	208 [207–219.50]	< 0.001
Number of SARS-CoV-2 vaccine doses received (*n* (%))			
- At least 1 dose	148 (100)	32 (100)	0.002
- At least 2 doses	148 (100)	32 (100)	
- At least 3 doses	147 (99.32)	31 (96.88)	
- At least 4 doses	110 (74.32)	15 (46.88)	
- At least 5 doses	35 (23.65)	0 (0)	
BTI infection after vaccination (*n* (%))			
- After second dose	4 (2.70)	0 (0)	0.347
- After third dose	44 (29.70)	5 (15.60)	0.104
- After fourth dose	21 (14.20)	1 (3.10)	0.083
- After fifth dose	12 (8.10)	0 (0)	0.515
Mean time of BTI (days; median [IQR])	208 [105.25–293.75]	56 [49.50–136]	0.045
Disease severity of BTI (*n* (%))			
- Asymptomatic	36 (24.32)	6 (18.80)	0.001
- Mild	33 (22.30)	0 (0)	
- Severe	12 (8.10)	0 (0)	
Death (*n* (%))	1 (0.68)	0 (0)	0.641

SOT, solid organ transplant; IC, immunocompetent; IQR, interquartile range; CNI, calcineurin inhibitors; TAC, tacrolimus; CsA, ciclosporin A; MMF, mycophenolate mofetil; MPS, mycophenolate sodium; mTOR-i, mTOR inhibitors; BTI, breakthrough infection. NA, Not applicable.

##### 2.1.1.1 SARS-CoV-2 vaccination

All patients tested negative for serology against nucleoprotein and spike SARS-CoV-2 antigens prior to the first vaccination and had no recorded history of a positive nasopharyngeal PCR swab. A total of 146 (81.11%) patients received the mRNA-1273 (Moderna Inc., Massachussetts, US) vaccine and 34 (18.89%) the BNT162b2 (Pfizer-BioNTech Inc, New York, US). The first two doses were administered between March and June 2021, the third between September and December 2021, and the fourth and fifth doses were given between April 2022 and May 2023. All patients in the study received three doses; however, one SOT and one IC patients received only two doses. Among the patients, 74.32% (110/148) SOT recipients and 46.88% (15/32) of IC recipients received four doses, while only 23.65% (32/148) of SOT recipients, and none of the IC recipients, received a fifth dose. All patients were followed up for at least 3 months after the fifth dose (July 2023) (**Table 1**).

##### 2.1.1.2 COVID-19 breakthrough infections

During the study period, all positive SARS-CoV-2 PCR tests were automatically reported to our regional Ministry of Health, ensuring accurate control of viral infection s. All patients were followed up by our different teams to record symptoms and clinical evolution. BTI occurred in four cases after the second dose, 49 cases after the third dose (44 [89.6%] SOT and 5 [10.4%] IC patients), 22 cases after the fourth dose (21 [95.45%] SOT and 1 [4.55%] IC), and 12 SOT patients after the fifth dose. BTI were classified according to World Health Organization guidelines as follows: severe if hospitalized with clinical signs of pneumonia plus oxygen supply requirements; mild when there is no evidence of viral pneumonia or hypoxia but showed COVID-19-related symptoms; and asymptomatic if only tested positive for SARS-CoV-2 by RT-PCR with no clinical or biological symptoms (e.g., lymphopenia, elevated PCR) (41). The main SARS-CoV-2 strain responsible for BTI after the second vaccine dose was predominantly the Delta variant, whereas all BTI following the third, fourth, and fifth doses were caused by Omicron subvariants, including BA.1, BA.2, BA.5, BQ. 1, and XBB.1 strains.

#### 2.1.2 Randomized controlled trial (Tor-vax)

In view of the results obtained from the previous study, we designed a multicenter randomized controlled interventional trial to assess the value of switching to a CNI/mTOR-i-based regimen from a CNI/MMF-based IS, to achieve protective immunity based on SARS-CoV-2 NAb and cellular immune memory responses after a fourth mRNA-based booster vaccination in previously unresponsive SOT (**Figure 1**) (Eudract 2022-000617-13). Patients were randomized in a 1:1 ratio into two groups: group A (control group: CNI/MMF) and group B (conversion group: CNI/mTOR-i). Randomization was stratified according to the type of transplanted organ and donor age. To qualify for recruitment, all candidates had to be receiving chronic maintenance immunosuppression with a CNI/MMF-based immunosuppressive regimen.

As illustrated in **Supplementary Figure S1**, of the 50 patients enrolled, 12 were infected right before randomization; thus 38/50 (76%) were randomized to remain on the same immunosuppressive regimen (group A; *n* = 17, 44.7%) or switch to a mTOR-i-based regimen (group B; *n* = 21, 55.3%). After receiving the fourth vaccine dose, nine patients from the mTOR-i group and four from the control group also dropped out, mainly because of COVID-19 BTI. Consequently, 25/50 (50%) of the SOT patients remained on protocol at month 1 after vaccination (T2a). Due to the high incidence of COVID-19 BTI during the recruitment process, the primary endpoint of the study could not be assessed with sufficient statistical power. Consequently, a descriptive analysis between groups of patients remaining on protocol was performed.

Inclusion and exclusion criteria are depicted in detail in the **Supplementary Materials**. Briefly, the trial included stable adult SOT recipients who had not IgG developed NAb against SARS-CoV-2 after three previous mRNA-based vaccine doses, had no previous COVID-19 infection (absence of anti-N IgG Ab), and had available baseline peripheral blood samples.

##### 2.1.2.1 Main endpoints of the study and sample size

The primary endpoint was to evaluate the capacity of each treatment group to achieve a protective serological immune response, defined as the development of SARS-CoV-2-specific IgG NAb, 1 month after receiving a fourth (T2a) SARS-CoV-2 mRNA-based vaccine. Secondary endpoints included comparing the percentage of seroconversion rates, frequencies of SARS-CoV- 2-specific IgG-producing mBc and cytokine-secreting T cells, and the number and severity of COVID-19 breakthrough infections between the two groups.

To detect a 20% superiority margin in NAb rates after switching to an mTOR-i-based immunosuppressive regimen, a total of 50 patients were needed to achieve statistical differences with an 80% statistical power, including 10% drop-out rates.

##### 2.1.2.2 Inclusion criteria

Clinically stable adult SOT recipients, at least 12 months after transplantation, from two different transplant centers in Barcelona, Spain (Vall d’Hebron and Bellvitge University Hospitals), who lacked IgG NAb against SARS-CoV-2 after receiving three previous mRNA-based vaccine doses, were eligible to participate.

##### 2.1.2.3 Exclusion criteria

Exclusion criteria included the presence of NAb against SARS-CoV-2, unavailability of baseline peripheral blood samples, previous COVID-19 infection (absence of anti-N IgG Ab), previous diagnosis of T-cell or antibody-mediated rejection within the previous 2 years, and the presence of donor-specific-HLA antibodies.

### 2.2 Collection and management of biological samples

Peripheral blood mononuclear cells (PBMC) and serum samples were obtained at the following specific time points for the descriptive cohort: prevaccination (T0), after the first dose (T1: 28 days; interquartile range [IQR], 28–34), 2 months after second dose (T2: 89 days; IQR, 79.25–95.75), 5 months after second dose (T3: 189 days; IQR 182–196), and 1 month after the third dose (T4: 232 days; IQR, 220–244).

In the randomized controlled trial whole blood and serum samples were obtained at the following prespecified time-points: at the time of randomization (T0a) (being one month prior to the fourth booster vaccination); at the time of the fourth dose (in case of Group B patients, being 1 month after mTOR-i conversion) (T1a) and at 1 month after fourth dose (T2a).

Peripheral blood mononuclear cells (PBMCs) were isolated from the patient’s blood by Ficol density gradient centrifugation and subsequently frozen in liquid nitrogen until their use in functional analyses. Serum samples were isolated by centrifugation and stored at – 20°C.

### 2.3 Assessment of SARS-CoV-2-specific adaptive immune memory

#### 2.3.1 SARS-CoV-2-specific humoral memory

##### 2.3.1.1 SARS-CoV-2-specific serological memory

###### 2.3.1.1.1 SARS-CoV-2-specific serum IgG antibodies

SARS-CoV-2-specific IgG antibodies were assessed against two main SARS-CoV-2 antigens using two ELISA platforms (Elecsys anti-SARS-CoV-2 for nucleoprotein and LIAISON Sars-CoV-2 TrimericS for spike glycoprotein). Detailed information on the methodology and interpretation is provided in the **Supplementary Material**. Antinucleoprotein IgG antibodies were assessed only at T0 to ensure that patients had not been previously infected.

###### 2.3.1.1.2 SARS-CoV-2-specific neutralizing antibodies

Neutralizing antibodies against the spike protein of SARS-CoV-2 from the Wuhan (D614G variant) and Omicron (BA.5 variant) strains were assessed using a plasma neutralization assay with a pseudotyped VSV-SARS-CoV-2 spike expressing luciferase (VSV-ΔG-Luc-S) (42). Detailed information on the methodology and interpretation is provided in the **Supplementary Material**.

As illustrated in **Supplementary Figure S1**, we confirmed previous reports (43, 44) showing that a cutoff of 143 BAU/mL for anti- spike IgG titers is an appropriate threshold for differentiating between patients with high antiviral neutralization activity. Notably, IgG levels and neutralizing antibody activity showed a positive correlation of *R* = 0.411 (*p* = 0.046).

##### 2.3.1.2 RBD-specific spike IgG-producing memory B cells

Circulating SARS-CoV-2-specific IgG-producing mBc frequencies were assessed against the receptor-binding domain of SARS-CoV-2 spike (RBD-S) protein using a colorimetric B-cell ELISPOT assay (45). A thorough description of the method of this assay is depicted in the **Supplementary Material**.

#### 2.3.2 SARS-CoV-2-reactive cytokine-producing memory T cells

Five distinct cytokine-producing Th1 and Th2 T-cell frequencies were simultaneously detected at all time points: effector (IFN-γ), proliferative (IL-2), central (IFN-γ/IL-2) Th1, proliferative (IL-21) Th2 and Th17, and stimulating (IL-5) Th2. Precise information is depicted in the **Supplementary Methods** in **Supplementary Data Sheet 1**.

#### 2.3.3 Analysis of immune memory compartments

We also assessed and defined the response of the two main immune memory compartments in a qualitative manner: the B-cell compartment, which includes serological NAb and memory B-cell responses, and the Th1 T-cell compartment, which includes IFN-γ and/or IL-2 T-cell responses. Further interpretation of the different responses is provided in the **Supplementary Material**.

### 2.4 Statistics

All statistical analyses were performed using SPSS version 26 software and R package version 1.0.12, and graphs were generated using GraphPad Prism version 8.0 software (GraphPad Software, San Diego, CA, USA). Continuous variables were expressed as median [IQR] and categorical variables as number of total (*n*) and percentage (%). Comparison s between groups was performed using Pearson’s *χ* ^2^ test for categorical data. Continuous measurements were compared among groups using the Kruskal–Wallis and Mann–Whitney *U* tests for nonnormally distributed data, and ANOVA and *t*-tests were used when data were normally distributed. *p*-values of < 0.05 were considered statistically significant. To assess longitudinal data, considering the missing values and the changes over time, a two/linear mixed model, considering study time points and transplant status as fixed effects, was used. A correlation analysis of repeated measurements (46) was performed. *p*-values were adjusted for multiple comparisons using the Benjamini–Hochberg method. Univariate logistic regression models were used to investigate the influence of clinical and immunological covariates by means of odds ratios (OR) with 95% confidence intervals.

## 3 Results

### 3.1 Patients and outcomes of the observational cohort

The first observational cohort consisted of 148 SOT patients who were compared to 32 IC individuals regarding serological and cellular immune memory responses triggered after three consecutive doses of SARS-CoV-2 mRNA-based vaccine s (**Figure 1**). Most patients were kidney (37%) and liver (28%) transplant recipients, receiving CNI-based immunosuppressive therapy combined with either MMF/MPS (*n* = 84) or an mTOR-i drug (*n* = 20). All patients received at least three booster doses. Among the 148 SOT patients, 110 (74.3%) received a fourth dose and 34 (23.7%) received a fifth booster dose. All patients were followed up for a median of 30 months (median, 892 days; IQR, 884–903) (**Table 1**). While COVID-19 BTI occurred after the third dose in 27.5% of patients (29.9% of SOT and 16.1% of IC patients) and in 17.6% of those who received a fourth dose (19.1% SOT and 6.7% IC), up to 34.3% of SOT patients who received a fifth dose developed COVID-19. Severe and mild COVID-19 only occurred among SOT in 12 (8.1%) and 33 (22.3%) patients, respectively. Asymptomatic COVID-19 was reported in 42 patients (36 (24.3%) SOT and six (18.8%) IC).

### 3.2 Strength and kinetics of adaptive immune memory after booster vaccination

**Figure 2:**
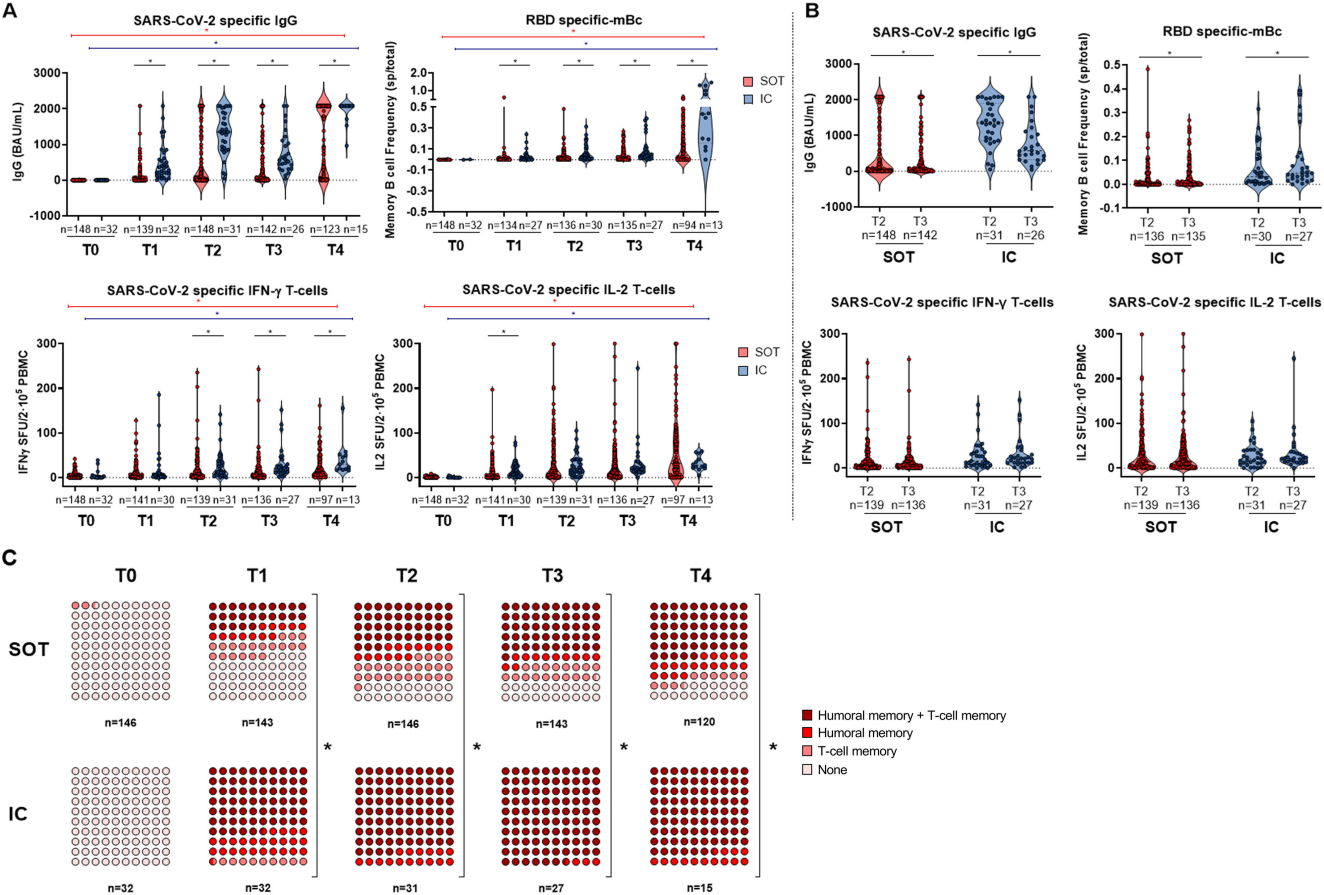
**(A)** SARS-CoV-2-specific serological and B- and T-cell memory immune responses between SOT and IC over time before and after booster vaccination. Significant intra- and intergroup differences are shown in **Supplementary Tables S1**, **S2**. **(B)** SARS-CoV-2-specific serological and memory-cell differences at 3 months postvaccination (two doses) and at 6 months postvaccination (two doses). **(C)** Global qualitative SARS-CoV-2-specific immune responses at main memory compartments between SOT and IC over time after booster vaccination. SOT, solid organ transplant; IC, immunocompetent; mBc, memory B cell; IFN-γ, interferon gamma; IL, interleukin; SFU, spot forming unit. ^*^ *p* < 0.05.

As illustrated in **Figure 2A**, while IgG titers, as well as mBc and T-cell frequencies specific to SARS-CoV-2, significantly increased with the number of vaccine doses in both groups, SOT patients displayed significantly weaker immune memory responses compared to IC patients after each vaccine dose, except for Th1 cells producing IL-2 and Th2 cells producing IL-21 and IL-5 (**Supplementary Tables S1**, **S2**). After the second dose, both at T2 and T3, 41.9% (62/148) and 38.7% (55/142) of SOT patients developed NAb, whereas up to 96.8% (30/31) and 92.3% (24/26) of IC patients did, respectively. Notably, after the third dose (T4), 100% (15/15) of IC patients showed NAb, while only 67.7% (84/124) of SOT patients were NAb positive. There was generally a strong positive correlation between all distinct SARS-CoV-2-specific immune responses except for mBc and T-cell frequencies between T0 and T4 (**Supplementary Table S3**).

Notably, within the 3-month time frame between the second and third vaccine doses (T2 and T3), no major changes were observed in all SARS-CoV-2-specific T memory responses, and the antibody titers progressively decayed. However, RBD-specific IgG-producing mBc significantly increased over time in both SOT and IC individuals (**Figure 2B**).

When both B- and T-cell immune compartments were assessed together, IC individuals showed earlier detection of memory responses across all compartments compared to SOT patients (**Figure 2C**). Specifically, after two vaccine doses (T3), most IC patients (27/31; 87.1%) displayed broad immune memory responses, whereas only 45.2% (66/146) of SOT patients did (*p* < 0.001). Similarly, after the third dose (T4), only 63.6% (75/118) of SOT patients showed complete detectable immune responses, while this protection was seen in all IC patients (*p* < 0.001). Importantly, at this last time point, up to 15.3% (18/118) of SOT patients did not show any immune response, 11.9% (14/118) showed responses only within the T-cell immune compartment, and 9.3% (11/118) showed responses only within the humoral compartment.

### 3.3 Neutralizing cross-reactivity of serum and IgG-producing RBD-specific mBc

**Figure 3:**
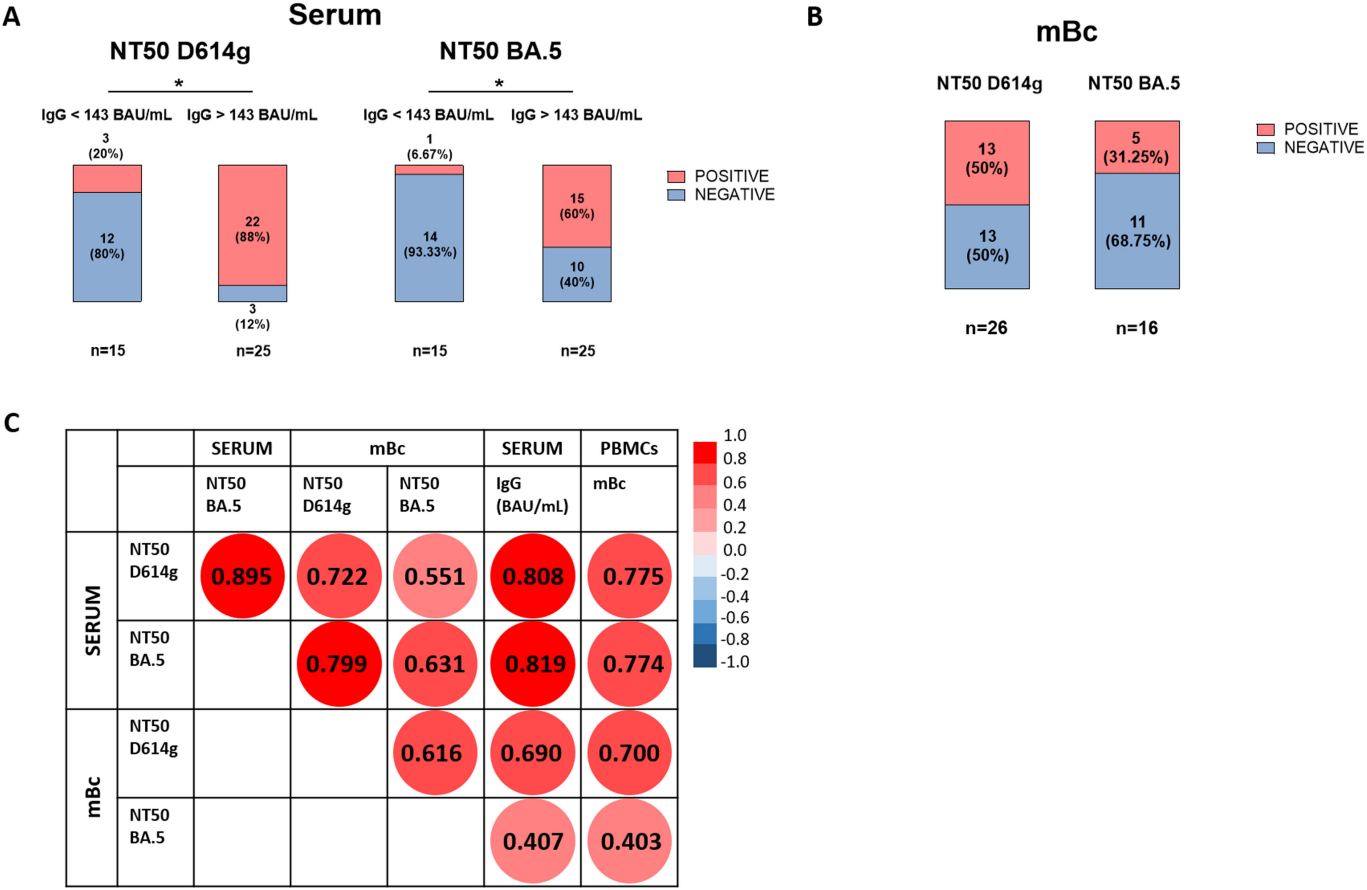
Neutralizing antibodies against SARS-CoV-2 in serum and supernatant samples of both SOT and IC patients. **(A)** Presence of neutralizing antibodies against Wuhan D614g and Omicron BA.5 strains in serum samples of patients with IgG levels < 143 BAU/mL and > 143 BAU/mL. ^*^ *p* < 0.05. **(B)** Presence of neutralizing antibodies against Wuhan D614g and Omicron BA.5 strains in supernatant samples of patients with detectable NAb in serum. **(C)** Spearman correlations between IgG levels in serum, frequencies of mBc, and neutralization titers against Wuhan D614g and Omicron BA.5 variants in both serum and supernatant from mBc samples. All correlations were statistically significant at *p* < 0.05.

In all patient cohorts, a positive correlation between serum IgG titers and the frequencies of IgG-producing mBc was observed in both SOT and IC individuals at all time points of assessment (**Supplementary Figure S2**). We next assessed the presence of NAb against two different viral strains, Wuhan (D614g) and Omicron (BA.5), to evaluate the cross-reactivity of both serum IgG and those produced by mBc in SOT and IC patients after four vaccine doses. As shown in **Figure 3A**, 88% (22/25) of patients with high IgG titers (> 143 BAU/mL) showed NAb in the serum against the Wuhan strain, whereas only 20% (3/15) of patients with low IgG titers (< 143 BAU/mL) did. Similar results were observed for the Omicron BA.5 variant, with 60% (15/25) of patients with high IgG titers showing NAb, compared to only 6.7% (1/15) of patients with low titers. Of note, the assessment of the neutralizing capacity of IgG antibodies produced by polyclonally expanded mBc revealed that 50% (13/26) and 31.3% (5/16) of patients showed mBc with NAb against the Wuhan and the Omicron BA.5 variants, respectively (**Figure 3B**). As shown in **Figure 3C**, there was a high correlation between serum and mBc NAb for both viral strains.

### 3.4 Immunosuppression regimens and immune memory after vaccination

**Figure 4:**
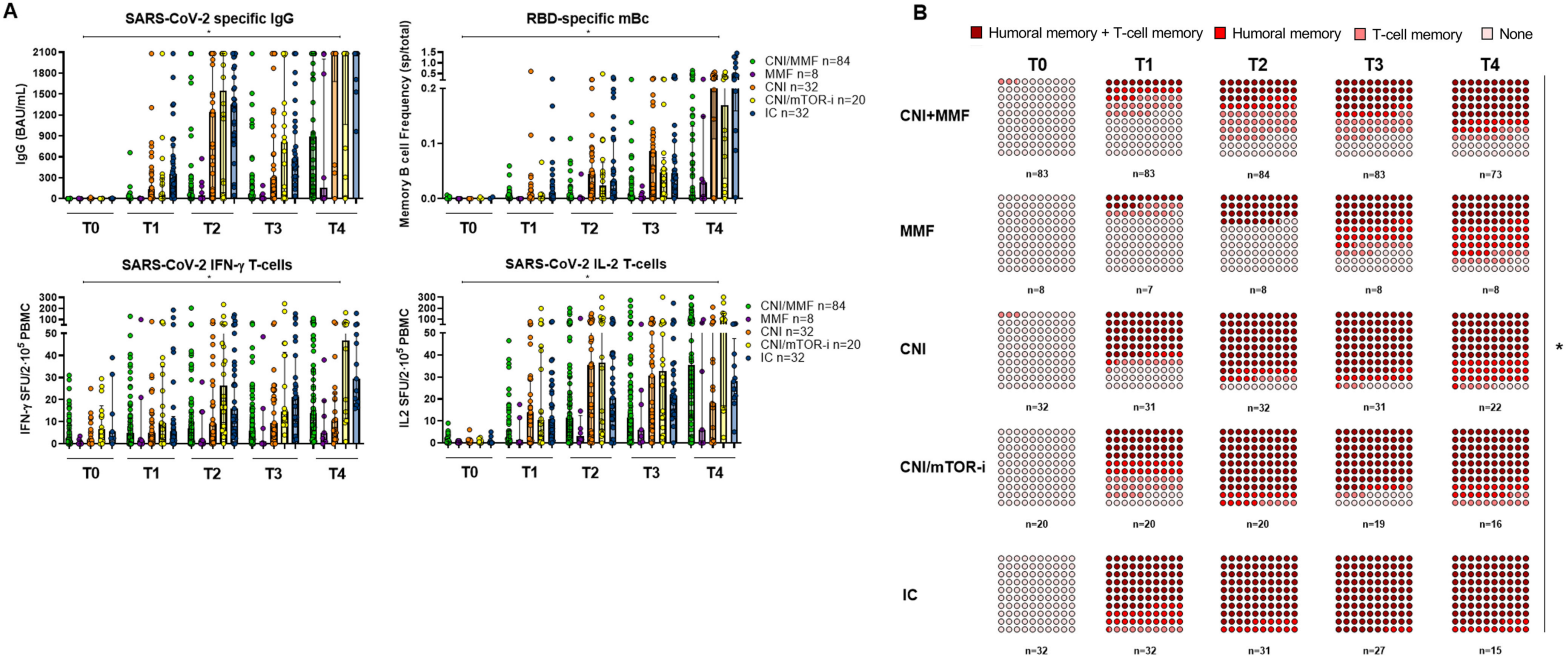
**(A)** SARS-CoV-2-specific serological and B- and T-cell memory immune responses between SOT with different IS protocols and IC over time before and after booster vaccination. Significant intra- and intergroup differences are shown in **Supplementary Table S4**. **(B)** Global qualitative SARS-CoV-2-specific immune responses at main memory compartments between SOT with different IS regimes and IC over time after booster vaccination. Significant intra- and intergroup differences are shown in **Supplementary Table S5**. ^*^ *p* < 0.05 is referred to the comparison made between all study groups.

Distinct maintenance immunosuppressive (IS) regimens were associated with different humoral and cellular immune responses (**Figure 4A**). Patients on CNI monotherapy and those on a mTOR-i-based regimen displayed significantly higher immune responses after each vaccine dose, comparable to those of IC individuals. In contrast, patients on CNI/MMF and those few on MMF monotherapy showed the weakest responses, even after three vaccine doses (**Supplementary Table S4**). When both B- and T-cell immune memory compartments were analyzed together, patients on either CNI monotherapy or CNI/mTOR-i immunosuppression developed a global immune response significantly faster than those on CNI/MMF and MMF monotherapy (**Figure 4B**; **Supplementary Table S5**).

### 3.5 RBD-specific memory B cells predict NAb seroconversion after booster vaccination and protection from severe COVID-19

**Figure 5:**
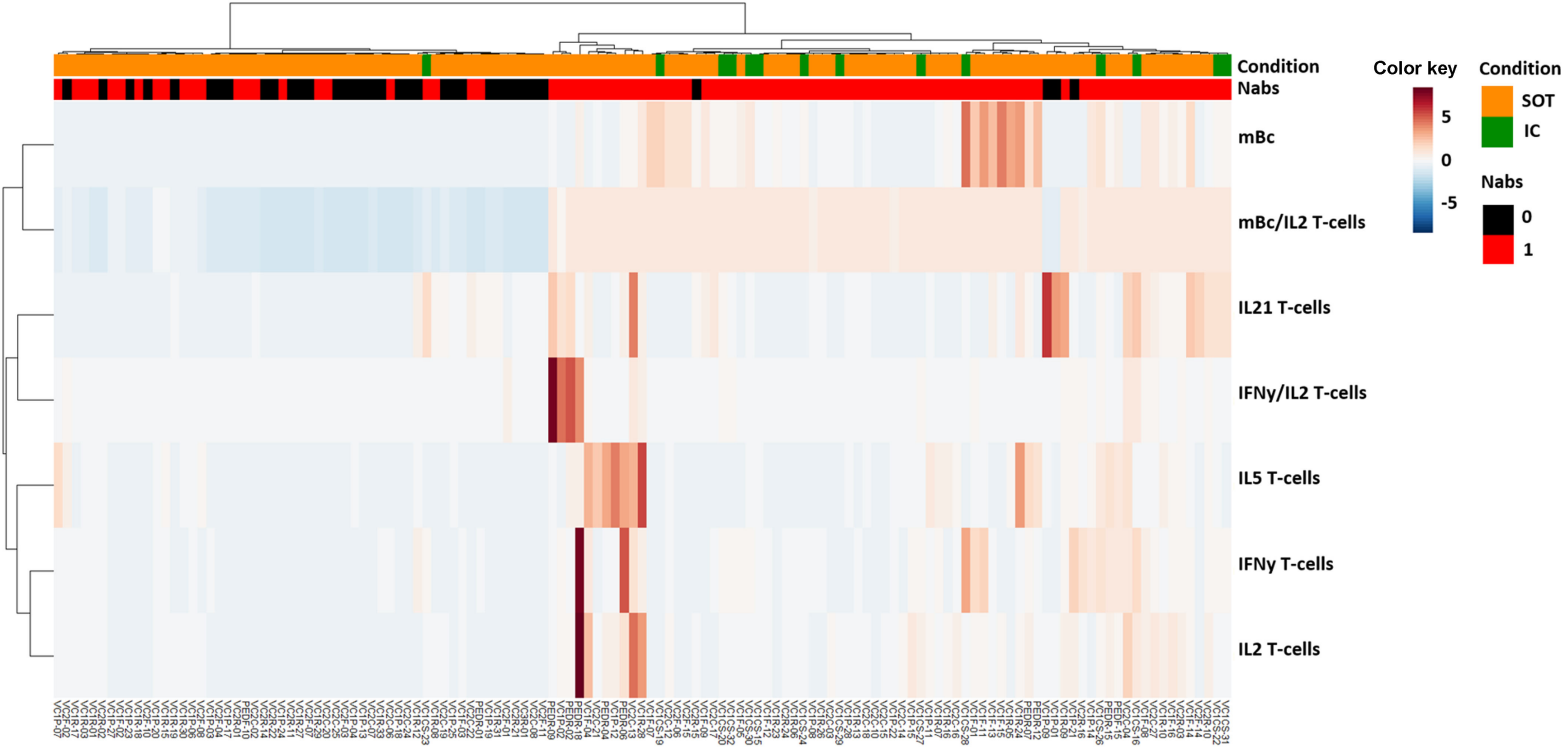
Heatmap generated by hierarchical clustering of different SARS-CoV-2-specific memory immune responses for SOT and IC patients, according to the development of SARS-CoV-2-specific neutralizing antibodies (NAb).

**Figure 6:**
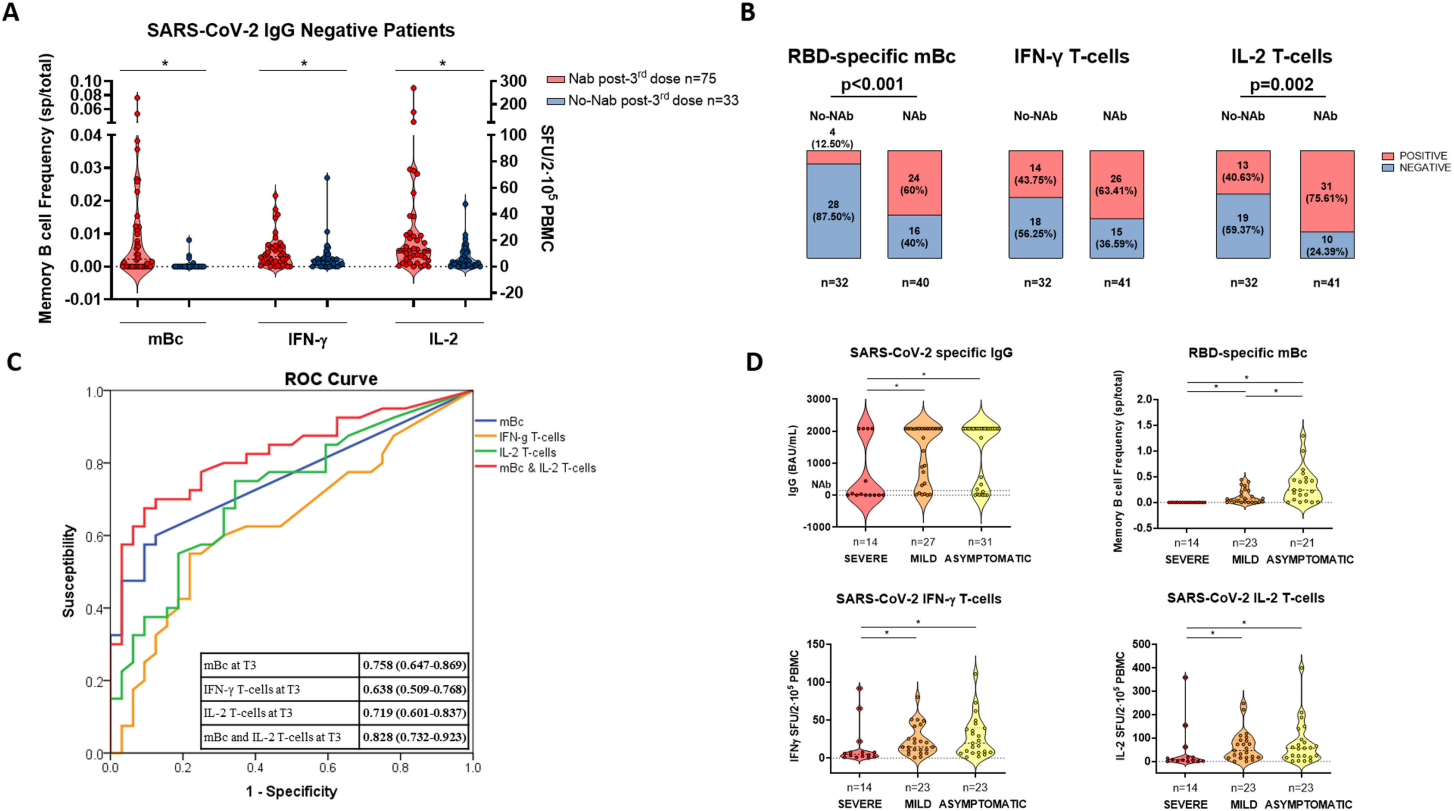
SARS-CoV-2-specific immune-memory responses predicting NAb and severity of breakthrough infection after third booster vaccination. **(A)** Pre- third vaccine dose frequencies of SARS-CoV-2-specific memory B and T cells in patients developing or not NAb after third booster vaccination in previously seronegative patients. **(B)** The presence of pre- third dose vaccine of SARS-CoV-2-specific memory T- and B-cell responses between patients developing or not NAb after third vaccination in seronegative patients. **(C)** ROC curve of SARS-CoV-2-specific immune-memory responses predicting the advent of NAb after vaccination in seronegative patients. **(D)** SARS-CoV-2-specific serological and memory-cell immune responses in patients developing distinct severity of SARS-CoV-2 breakthrough infection. Significant intra- and intergroup differences are shown in **Supplementary Table S7**. mBc, memory B cell; IFN-γ, interferon gamma; IL, interleukin; SFU, spot forming unit. ^*^ *p* < 0.05.

Unsupervised hierarchical cluster analysis of all immune memory compartments prior to the third booster vaccination in all patients showed that the presence of prebooster cellular immune memory, especially RBD-specific IgG-producing mBc, predicted the successful development of NAb after the booster vaccination (**Figure 5**; **Supplementary Table S6**). Similarly, when only seronegative patients were analyzed (**Figures 6A, B**), those who developed NAb after a third booster vaccination displayed significantly higher IFN-γ, IL-2, and mBc frequencies prior to vaccination. Among these, RBD-specific mBc frequencies showed the best predictive capacity for the subsequent development of NAb (AUC, 0.758) (**Figure 6C**). Moreover, the combination of SARS-CoV-2-specific IL-2-producing T cells and RBD-specific mBc provided the best prediction for subsequent NAb (AUC, 0.828) development.

Multivariate analysis revealed that the main factors favoring the development of NAb were the presence of RBD-specific mBc (OR, 19.313 [95% CI, 3.721–100.24]; *p* < 0.001), lower age (OR, 0.920 [95% CI, 0.863–0.980]; *p* = 0.010), and CNI trough levels (OR, 0.771 [95% CI, 0.586–1.013]; *p* = 0.062). Similarly, for the development of RBD-specific mBc, the time after transplant (OR, 1.007 [95% CI, 1.002–1.013]; *p* = 0.012) and the absence of MMF-based therapy—with either the combination of CNI/mTOR-i (OR, 11.092 [95% CI, 2.084–59.029]; *p* = 0.005) or CNI monotherapy (OR, 4.224 [95% CI, 1.482–12.040]; *p* = 0.007)—were independent correlates.

When we analyzed the association between memory immune responses and subsequent COVID-19 BTI during the study period, significantly lower cytokine-producing T-cell frequencies and a lack of RBD-specific mBc were observed among patients who developed severe COVID-19 compared to those with mild or asymptomatic infections (**Figure 6D**; **Supplementary Table S7**). Multivariate analysis, considering both clinical and immunological variables predicting severe COVID- 19, showed that the only factor favoring severe BTI was the absence of RBD-specific mBc (OR, 0.097 [95% CI, 0.018–0.533]; *p* = 0.007) (**Supplementary Table S8**).

### 3.6 RBD-specific mBC and changes in immunosuppression favor successful immunization in nonresponders

A total of 25 SOT patients who did not develop NAb despite receiving three consecutive vaccine doses were subsequently evaluated in a RCT. This trial compared standard-of-care CNI/MMF immunosuppression (group A) with a low-exposure CNI/mTOR-i-based regimen (group B) to assess the development of anti-SARS-CoV-2 protective immunity at 1 month after the fourth vaccine dose (**Supplementary Figure S1**). The two groups did not differ in terms of main clinical, demographic, or immunological baseline characteristics (**Table 2**).

**Table 2:** Main demographic and clinical characteristics of the randomized controlled trial population.

Patients of the randomized controlled trial (*N* = 38)	Group A: CNI/MMF (*N* = 17)	Group B: CNI/mTOR-i (*N* = 21)	*p*-value
Age (years; median [IQR])	69 [58–73]	66 [58.50–70]	0.269
Sex (female; *n* (%))	8 (47.10)	10 (47.60)	0.973
Time after transplant (months, median [IQR])	53 [21.18–99.55]	41.50 [30.48–124.94]	0.758
Organ (*n* (%))			
- Kidney	11 (64.70)	13 (61.90)	0.686
- Lung	5 (29.40)	5 (23.80)	
- Heart	1 (5.90)	3 (14.30)	
Induction treatment (*n* (%))			
- No	2 (11.80)	3 (14.30)	0.211
- Anti-thymocyte globulins	5 (29.40)	6 (28.60)	
- Anti-CD25	3 (17.60)	9 (42.90)	
- Others	7 (41.20)	3 (14.30)	
Maintenance steroids (*n* (%))	16 (94.10)	20 (95.20)	0.878
- Steroids dose (mg/day; median [IQR])	5 [5–8]	5 [5–5]	0.253
Main comorbidities (*n* (%))			
- Diabetes	7 (41.20)	14 (66.70)	0.116
- Arterial hypertension	9 (52.90)	16 (80)	0.080
- Obesity	3 (17.60)	4 (19)	0.912
- Active neoplasm	0 (0)	(0)	–
Time of immune monitoring before and after vaccination (days; median [IQR])			
- T0a (*n* = 38)	0 [0–0]	0 [0–0]	1.000
- T1a (*n* = 19)	NA	31 [27–34]	–
- T2a (*n* = 25)	45 [38–50.50]	59 [55–73]	0.003
CNI trough (ng/mL; median [IQR])			
- T0a (*n* = 38)	7.50 [5.70–8.50]	7.35 [4.93–9.98]	1.000
- T1a (*n* = 19)	NA	5.50 [4.05–6.70]	–
- T2a (*n* = 25)	7 [6.10–8.70]	5.30 [4.05–6.70]	0.013
Sirolimus trough (ng/mL; median [IQR])			
- T0a (*n* = 38)	NA	NA	–
- T1a (*n* = 19)	NA	5 [4.15–7.75]	–
- T2a (*n* = 25)	NA	5.65 [4.73–11.15]	–
Baseline serological immunity			
- NAb (*n* (%))	0 (0)	0 (0)	–
- IgG titers (BAU/mL; median [IQR])	4.81 [4.81–55.69]	9.67 [4.81–50.53]	0.558
Sirolimus discontinuation (%)			
- Before T2a (month 1)	NA	2 (9.52)	–
- After T2a (month 6)	NA	4 (19.05)	–
BTI infection (%)			
- Before T2a (month 1)	5 (29.42)	4 (19.05)	0.304
- After T2a (month 6)	3 (17.65)	4 (19.05)	0.578
Disease severity of BTI (*n* (%))			
- Asymptomatic	4 (23.50)	1 (4.80)	0.370
- Mild	3 (17.60)	6 (28.60)	
- Severe	1 (5.90)	1 (4.80)	
Death (*n* (%))	1 (5.88)	0 (0)	0.989

SOT, solid organ transplant; IC, immunocompetent; IQR, interquartile range; CNI, calcineurin inhibitors; TAC, tacrolimus; CsA, ciclosporin A; MMF, mycophenolate mofetil; MPS, mycophenolate sodium; mTOR-i, mTOR inhibitors; BTI, breakthrough infection.

NA, Not applicable.

**Figure 7:**
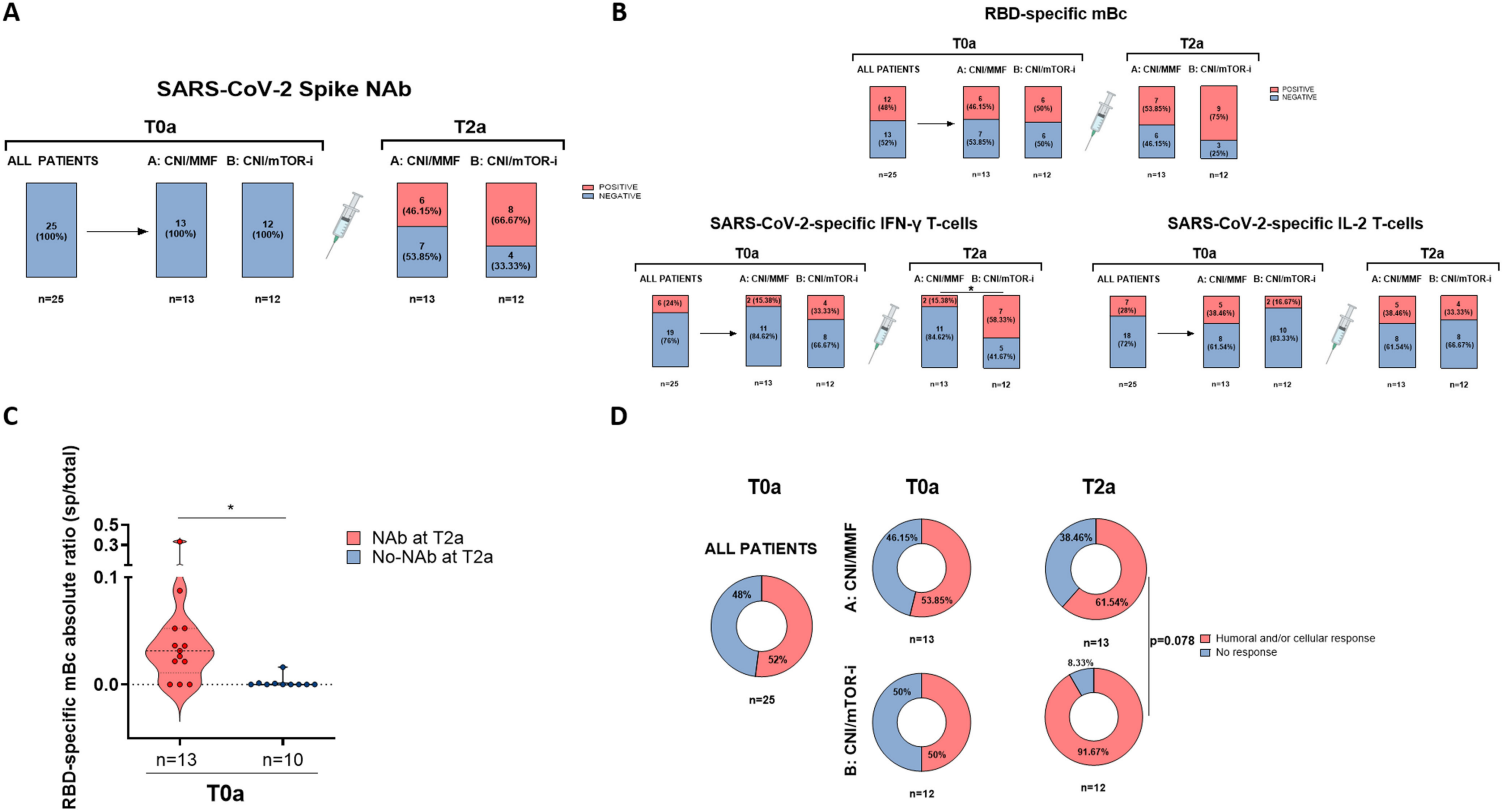
Results of the randomized controlled trial. **(A)** Presence of SARS-CoV-2-specific NAb (main end-point of the study) in patients from both groups of the trial after the fourth vaccine dose (T2a). **(B)** Presence of RBD-specific memory B - and memory T-cell responses in patients from both groups of the trial after the fourth vaccine dose (T2a). **(C)** Frequencies of RBD-specific mBc prior to the fourth dose (T0a) in patients with and without neutralizing antibodies after the fourth vaccine dose (T2a). Patients from both groups **(A, B)** were combined according to the presence of neutralizing antibodies at T2a. **(D)** Global qualitative SARS-CoV-2-specific immune responses at main memory compartments for both groups of study **(A, B)** before (T0a) and after the fourth dose (T2a). Humoral and/or cellular response means the presence of either NAb, mBc, and/or IFN-γ/IL-2 T-cells. ^*^ *p* < 0.05.

Firstly, although none of the 12 CNI/mTOR-i patients developed NAb before the fourth vaccine booster, we observed that cellular immune responses had already appeared 1 month after conversion to CNI/mTOR-i and prior to vaccination (T1a) in some patients (RBD-specific mBc, two patients; IFN-γ- and IL-2-producing T cells, two and one patients, respectively) (**Supplementary Figure S4**). Secondly, 1 month after vaccination (T2a), 20% more patients developed NAb in group B compared to group A (8/12 [66.7%] vs. 6/13 [46.1%], *p* = 0.306, respectively) (**Figure 7A**). Additionally, the proportion of patients with detectable SARS-CoV-2-specific IgG-, IFN- γ- and IL-2-producing B- and T-cell responses, respectively, was also numerically higher in group B than in group A at T2a (9/12 [75%] vs. 7/13 [53.8%], *p* = 0.271 for RBD-specific mBc; 7/12 [58.3%] vs. 2/13 [15.4%], *p* = 0.025 for IFN-γ; and 4/12 [33.3%] vs. 5/13 [38.5%], *p* = 0.790 for IL-2-producing T cells, respectively) (**Figure 7B**). Despite the absence of NAb at baseline (T0a), some patients in each group displayed cellular responses; RBD-specific mBc in 12/25 (48%), IFN-γ-producing T cells in 6/25 (24%), and IL-2-producing T cells in 7/25 (28%). Although no clinical, demographic, or immunological variables predicted NAb seroconversion, prevaccine RBD-specific mBc was a significant predictor (OR, 33 [95% CI, 2.909–374.31]; *p* = 0.005) (**Figure 7C**). Similarly, in the observational cohort, there was a high correlation between serum IgG titers and IgG-producing mBc both after vaccination and between prevaccine mBc and postvaccine IgG titers (**Supplementary Figure S5**).

The proportion of patients receiving broader adaptive immune responses achieved after booster vaccination (NAb, RBD-specific mBc, and/or IFN-γ/IL-2-producing T cells) was higher in those switched to CNI/mTOR-i immunosuppression compared to those remaining on CNI/MMF (11/12 [91.7%] vs. 8/13 [61.5%], *p* = 0.078, respectively) (**Figure 7D**).

## 4 Discussion

In this study, we describe in a large cohort of SOT undergoing multiple mRNA-based vaccinations the impaired magnitude and kinetics of both humoral and functional memory T and B-cell responses specific to SARS-CoV-2 and the key role of cross-reactive RBD-specific mBc predicting successful neutralizing humoral responses to booster vaccination as well as protecting from severe clinical forms of COVID-19. Furthermore, in an RCT, we show that while the presence of circulating RBD-specific mBc in unresponsive SOT patients is key to predicting the development of NAb, regardless of the type of immunosuppression, switching from CNI/MMF to low-exposure CNI/mTOR-i immunosuppression appears to promote broader SARS-CoV-2-specific immune responses with the involvement of more immune compartments.

The first data from our study confirmed that both SOT and IC individuals display a progressive and generalized increase in detectable antiviral immune responses across major peripheral immune compartments (47–51). However, SOT patients display significantly weaker and more delayed humoral and cellular immunity compared to IC patients, with up to 35% of SOT patients lacking any functional immune response (such as NAb, RBD-specific IgG-producing mBc, and IFN-y and IL-2-producing T cells) despite receiving three vaccine doses. Conversely, despite being significantly older, IC patients in our study developed strong, detectable immune responses. Interestingly, despite a progressive decay in detectable serological titers between 2 and 5 months after vaccination, memory T- and B-cell frequencies were maintained, with RBD-specific mBc increasing over time. This finding underscores the long-lasting expansion and survival of mBc, in addition to serum antibodies, following active immunization, even among SOT recipients.

Despite the strong correlation between all functional immune memory responses at each time point and over time, illustrating a close biological interplay between different adaptive immune compartments, we found that detectable frequencies of IL-2-producing T cells, and especially of RBD-specific IgG-producing mBc, played a preponderant role in predicting successful vaccine responses. Indeed, patients with high frequencies of RBD-specific mBc and IL-2-producing T cells, even without detectable serum IgG titers, were more likely to develop NAb after booster vaccination. Furthermore, the absence of detectable frequencies of RBD-specific mBc strongly associated with a higher risk of developing severe forms of COVID-19 if infected. In this regard, we recently reported the importance of these two memory cell responses in providing long-lasting immune protection after natural SARS-CoV-2 infection and in distinguishing patients with better clinical outcomes when developing severe forms of COVID-19 (29, 30). To note, similar frequencies of T-cell responses were detected after natural infection compared to active immunization, whereas for RBD-specific mBc responses, patients with natural infection showed lower frequencies of mBc compared to those receiving three doses of the SARS-CoV-2 vaccine. Therefore, monitoring these two functional cellular immune responses could help identify SOT patients who might benefit from additional booster vaccinations and those who m ay require different preventive strategies to avoid COVID-19.

The relevance of SARS-CoV-2-specific mBc in contributing to serological memory is also illustrated by the strong correlation between serum antiviral IgG titers and those produced by circulating mBc thus, strongly suggesting a direct contribution of the peripheral mBc compartment to serum NAb. Interestingly, we also observed similar neutralizing cross-reactivity of both serum IgG and IgG-producing mBc against two different SARS-CoV-2 strains, Wuhan (D614g) and Omicron (BA.5), produced in response to either the vaccine or a natural BTI.

The development of broad immune responses, including humoral and distinct memory T- and B-cell compartments, was directly challenged by the time between transplant surgery and vaccination as well as the types of immunosuppression used. MMF-based regimens were the most clearly impaired, while CNI/mTOR-i displayed the most robust immune responses. In this regard, a recent report showed that a temporary MMF withdrawal from a CNI/MMF-based regimen in seronegative kidney transplant patients resulted in seroconversion in 76% of patients and a marked increase of RBD-specific B cells and plasmablasts after a fourth booster vaccination (38). Importantly, while this therapeutic approach may be safe for low immunological risk SOT patients (52–54), such a strategy may not be feasible for those who are recently transplanted (16) or at a high immunological risk (55). Furthermore, the withdrawal of MMF did not allow for the expansion of SARS-CoV-2-specific CD4+ cells, suggesting the need for an alternative strategy to foster broader antiviral immune compartments. To overcome these limitations, we designed an RCT to evaluate the efficacy of switching to a low-exposure CNI/mTOR-i-based regimen, considering both the safety of this immunosuppressive strategy (56) and the reported immunomodulatory effects of mTOR-i in promoting antiviral and vaccine-induced immune responses in other clinical settings (39, 57, 58). Interestingly, despite the number of study patients being lower than expected due to high infection rates during enrollment, we found an overall increase in SARS-CoV-2-specific immune responses in patients switched to CNI/mTOR-i as compared to those who remained on CNI/MMF. Indeed, a significantly higher proportion of patients switched to CNI/mTOR-i developed IFN-γ-producing T cells and had numerically higher levels of NAb and RBD-specific IgG-producing mBc compared to those remaining on CNI/MMF. Of note, 4 weeks after switching to CNI/mTOR-i but prior to the booster vaccination, some patients displayed novel detectable cellular responses, suggesting an immunomodulatory effect of this immunosuppressive strategy based on mTOR-i favoring SARS-CoV-2 vaccine-induced immunity. We did not perform any analysis 1 month after randomization in the group of patients who remained on the same immunosuppressive regimen, as no intervention was done and they just received the fourth vaccine dose. Nevertheless, while some patients displayed detectable SARS-CoV-2-specific T- or B-cell responses despite no NAb, only the presence of RBD-specific mBc predicted the advent of NAb after the fourth booster vaccination, regardless of the type of immunosuppressive regimen.

Our study has some limitations. While the inclusion of distinct SOT in this study could be considered a confounding factor, it actually allowed us to show that differences in immune responses are predominantly driven by different immunosuppressive regimens rather than by the types of SOT. Unfortunately, the sample size of patients analyzed in the trial was smaller than expected due to COVID-19 infections during the recruitment process. However, the results observed in the first observational study together with the overall improved humoral and cellular immune responses in the CNI/mTOR-i group of the RCT, strongly support the validity of these findings.

In summary, our work provides new evidence regarding the key role of SARS-CoV-2-specific mBc in facilitating successful responses to booster vaccination and in providing immune protection against severe forms of COVID-19 in SOT recipients. These data may have relevant clinical implications, as monitoring these cellular immune responses could guide decision-making on the type of preventive strategy to follow —either pursuing additional booster vaccinations or establishing alternative immunosuppressive strategies, such as low-exposure CNI/mTOR-i to promote broader SARS-CoV 2-vaccine-induced immunity. Additional interventional RCTs are highly warranted to further validate our findings and to guide the selection of the most suitable and safe immunosuppressive strategy for developing protective immunity after booster vaccinations.

## Data Availability

The raw data supporting the conclusions of this article will be made available by the authors, without undue reservation.
