# Supplementary material for: Role of SARS-CoV-2-specific memory B cells promoting immune protection after booster vaccination in solid organ transplantation
Source: Front Immunol. 2024 Oct 8;15:1463769. doi: 10.3389/fimmu.2024.1463769 (PMC11493670; doi:10.3389/fimmu.2024.1463769)
Supplement: Supplementary file 1 [file DataSheet1.docx]

***SUPPLEMENTARY MATERIAL***

**Supplementary Table 1.** Differences between SARS-CoV2-specific immune responses between SOT and IC at different time points.

|  |  | **SOT** | **IC** | **P Value** |
| --- | --- | --- | --- | --- |
| **IgG Spike titers**  **(BAU/mL)** | **T0** | 0 [0-0] | 0 [0-0] | p=0.710 |
|  | **T1** | 0 [0-18.37] | 221.78 [130.39-446.70] | **p<0.001** |
|  | **T2** | 39.52 [0-417.70] | 1350 [815.50-1975] | **p<0.001** |
|  | **T3** | 37.10 [0-258] | 451 [236-572.49] | **p<0.001** |
|  | **T4** | 1939 [35.24-2080] | 2080 [2080-2080] | **p=0.005** |

|  |  | **SOT** | **IC** | **P Value** |
| --- | --- | --- | --- | --- |
| **IFN-γ T-cell (SFU/2·10^5^ PBMC)** | **T0** | 1 [0-7.13] | 2.50 [1.25-9.50] | p=0.093 |
|  | **T1** | 4.75 [1-12.25] | 7 [1.75-9.25] | p=0.506 |
|  | **T2** | 8.75 [2-19.63] | 11 [3.50-35.25] | **p=0.010** |
|  | **T3** | 8.25 [3.38-16.13] | 21 [15.75-23.75] | **p<0.001** |
|  | **T4** | 14.25 [6-40.13] | 36 [24.25-50.75] | **p=0.008** |

|  |  | **SOT** | **IC** | **P Value** |
| --- | --- | --- | --- | --- |
| **RBD-sp mBc ratio (sp/total)** | **T0** | 0 [0-0] | 0 [0-0] | p=0.554 |
|  | **T1** | 0 [0-0.004] | 0.012 [0-0.025] | **p<0.001** |
|  | **T2** | 0 [0-0.017] | 0.012 [0.002-0.054] | **p<0.001** |
|  | **T3** | 0 [0-0.012] | 0.046 [0.023-0.105] | **p<0.001** |
|  | **T4** | 0.003 [0-0.012] | 0.436 [0.195-1.280] | **p<0.001** |

|  |  | **SOT** | **IC** | **P Value** |
| --- | --- | --- | --- | --- |
| **IL-2 T-cell (SFU/2·10^5^ PBMC)** | **T0** | 0 [0-0.5] | 0 [0-0.4] | p=0.312 |
|  | **T1** | 3 [0-10.13] | 8.50 [5.50-17] | **p=0.001** |
|  | **T2** | 13.25 [3-48] | 11 [3.75-14.50] | p=0.438 |
|  | **T3** | 13.50 [2.88-36.50] | 23.50 [17-25.50] | p=0.082 |
|  | **T4** | 33.25 [8.13-94] | 28 [23-49.75] | p=0.684 |

|  |  | **SOT** | **IC** | **P Value** |
| --- | --- | --- | --- | --- |
| **IFN-γ&IL-2 T-cell (SFU/2·10^5^ PBMC)** | **T0** | 0 [0-0.50] | 0 [0-0] | p=0.112 |
|  | **T1** | 0 [0-1] | 1 [0.75-2.50] | **p=0.009** |
|  | **T2** | 1.50 [0-3.50] | 3.50 [0.50-5.75] | **p=0.007** |
|  | **T3** | 1 [0-4] | 5 [3.50-7] | **p<0.001** |
|  | **T4** | 2 [0.50-9] | 6.50 [4.50-9] | p=0.108 |

|  |  | **SOT** | **IC** | **P Value** |
| --- | --- | --- | --- | --- |
| **IL-21 T-cell (SFU/2·10^5^ PBMC)** | **T0** | 0.50 [0-3] | 0.50 [0-12.50] | p=0.436 |
|  | **T1** | 2 [0-11] | 2 [0.75-16.25] | p=0.438 |
|  | **T2** | 8 [1.50-43] | 15.50 [3.25-30.75] | p=0.783 |
|  | **T3** | 11 [2.50-32] | 32 [9.50-76.25] | p=0.473 |
|  | **T4** | 26 [5.50-104] | 25 [8.50-43.75] | p=0.077 |

|  |  | **SOT** | **IC** | **P Value** |
| --- | --- | --- | --- | --- |
| **IL-5 T-cell (SFU/2·10^5^ PBMC)** | **T0** | 0 [0-0.50] | 0 [0-1] | p=0.716 |
|  | **T1** | 0.50 [0-2.50] | 1.50 [0.75-4.25] | **p=0.010** |
|  | **T2** | 4 [0-16] | 2 [0.50-23.50] | p=0.343 |
|  | **T3** | 5 [1-14] | 5 [1.25-7.50] | p=0.704 |
|  | **T4** | 7.50 [1-35] | 14.50 [8-39.75] | p=0.339 |

**Supplementary Table 2.** Evolution of SARS-CoV2-specific immune responses over-time. Columns *Time* show the evolution of immune responses over-time for all patients and *Time*SOT* the evolution of the immunological responses over-time considering the condition of being transplanted or not.

| **Variable** | **T0 - T3** | | **T0 - T4** | |
| --- | --- | --- | --- | --- |
|  | **Time** | **Time * SOT** | **Time** | **Time * SOT** |
| **NAb** | **<0.0001** | **<0.0001** | **<0.0001** | **<0.0001** |
| **RBD-sp mBc** | **<0.0001** | **0.0003** | **0.0033** | **<0.0001** |
| **IFN-g T-cells** | **<0.0001** | **0.0226** | **<0.0001** | **0.0191** |
| **IL-2 T-cells** | **<0.0001** | 0.5842 | **<0.0001** | 0.1232 |
| **IL-21 T-cells** | **<0.0001** | 0.5842 | **<0.0001** | 0.0721 |
| **IL-5 T-cells** | **<0.0001** | 0.2927 | **<0.0001** | 0.1172 |
| **IFN-g/IL-2 T-cells** | **<0.0001** | 0.8945 | **<0.0001** | 0.9508 |

| **Variable** | **T0 – T1** | | **T1 – T2** | | **T2 – T3** | | **T3 – T4** | |
| --- | --- | --- | --- | --- | --- | --- | --- | --- |
|  | **Time** | **Time * SOT** | **Time** | **Time * SOT** | **Time** | **Time * SOT** | **Time** | **Time * SOT** |
| **NAb** | **<0.0001** | **<0.0001** | **<0.0001** | **0.022** | **<0.0001** | **0.0004** | **<0.0001** | **0.0435** |
| **RBD-sp mBc** | **0.002** | 0.0579 | **0.002** | 0.2159 | **0.0083** | 0.2159 | **0.002** | **<0.0001** |
| **IFN-g T-cells** | **0.0001** | 0.1809 | **0.0055** | 0.6347 | 0.3016 | 0.5166 | **0.001** | 0.6347 |
| **IL-2 T-cells** | **<0.0001** | 0.1755 | **<0.0001** | 0.1754 | 0.4931 | 0.1801 | **<0.0001** | 0.1755 |
| **IL-21 T-cells** | **<0.0001** | 0.6028 | **0.0027** | 0.6816 | 0.1621 | 0.1391 | **0.0002** | 0.0542 |
| **IL-5 T-cells** | **<0.0001** | 0.2829 | **<0.0001** | 0.1519 | 0.0741 | 0.9421 | **0.0003** | 0.3021 |
| **IFN-g/IL-2 T-cells** | **<0.0001** | **0.0477** | **<0.0001** | 0.6689 | 0.2397 | 0.9143 | 0.8492 | 0.9196 |

**Supplemental Table 3.** Correlation between different SARS-CoV2-specific immune responses over time.

| **Variables** | | **T0 – T3** | | **T0 – T4** | |
| --- | --- | --- | --- | --- | --- |
|  |  | **R** | **p-value** | **R** | **p-value** |
| **NAb** | **mBc** | **0.4055** | **<0.0001** | **0.1621** | **0.0015** |
|  | **IFN-γ T-cells** | **0.3931** | **<0.0001** | **0.4058** | **<0.0001** |
|  | **IL-2 T-cells** | **0.4809** | **<0.0001** | **0.5325** | **<0.0001** |
|  | **IL-21 T-cells** | **0.2353** | **<0.0001** | **0.3409** | **<0.0001** |
|  | **IL-5 T-cells** | **0.3995** | **<0.0001** | **0.4226** | **<0.0001** |
|  | **IFN-g/IL-2 T-cells** | **0.279** | **<0.0001** | **0.2909** | **<0.0001** |
| **RBD-sp mBc** | **NAb** | **0.4055** | **<0.0001** | **0.1621** | **0.0015** |
|  | **IFN-γ T-cells** | **0.1823** | **0.0011** | 0.0592 | 1 |
|  | **IL-2 T-cells** | **0.2193** | **<0.0001** | 0.0393 | 1 |
|  | **IL-21 T-cells** | 0.1149 | 0.2304 | 0.0047 | 1 |
|  | **IL-5 T-cells** | **0.1507** | **0.0174** | 0.0547 | 1 |
|  | **IFN-g/IL-2 T-cells** | 0.1299 | 0.0889 | 0.0338 | 1 |
| **IFN-γ T-cells** | **NAb** | **0.3931** | **<0.0001** | **0.4058** | **<0.0001** |
|  | **mBc** | **0.1823** | **0.0011** | 0.0592 | 1 |
|  | **IL-2 T-cells** | **0.6272** | **<0.0001** | **0.6446** | **<0.0001** |
|  | **IL-21 T-cells** | **0.3237** | **<0.0001** | **0.3997** | **<0.0001** |
|  | **IL-5 T-cells** | **0.2746** | **<0.0001** | **0.3046** | **<0.0001** |
|  | **IFN-g/IL-2 T-cells** | **0.3883** | **<0.0001** | **0.4217** | **<0.0001** |
| **IL-2 T-cells** | **NAb** | **0.4809** | **<0.0001** | **0.5325** | **<0.0001** |
|  | **mBc** | **0.2193** | **<0.0001** | 0.0393 | 1 |
|  | **IFN-γ T-cells** | **0.6272** | **<0.0001** | **0.6446** | **<0.0001** |
|  | **IL-21 T-cells** | **0.4768** | **<0.0001** | **0.6424** | **<0.0001** |
|  | **IL-5 T-cells** | **0.5894** | **<0.0001** | **0.6294** | **<0.0001** |
|  | **IFN-g/IL-2 T-cells** | **0.5122** | **<0.0001** | **0.5136** | **<0.0001** |
| **IL-21 T-cells** | **NAb** | **0.2353** | **<0.0001** | **0.3409** | **<0.0001** |
|  | **mBc** | 0.1149 | 0.2304 | 0.0047 | 1 |
|  | **IFN-γ T-cells** | **0.3237** | **<0.0001** | **0.3997** | **<0.0001** |
|  | **IL-2 T-cells** | **0.4768** | **<0.0001** | **0.6424** | **<0.0001** |
|  | **IL-5 T-cells** | **0.3491** | **<0.0001** | **0.4505** | **<0.0001** |
|  | **IFN-g/IL-2 T-cells** | **0.354** | **<0.0001** | **0.3176** | **<0.0001** |
| **IL-5 T-cells** | **NAb** | **0.3995** | **<0.0001** | **0.4226** | **<0.0001** |
|  | **mBc** | **0.1507** | **0.0174** | 0.0547 | 1 |
|  | **IFN-γ T-cells** | **0.2746** | **<0.0001** | **0.3046** | **<0.0001** |
|  | **IL-2 T-cells** | **0.5894** | **<0.0001** | **0.6294** | **<0.0001** |
|  | **IL-21 T-cells** | **0.3491** | **<0.0001** | **0.4505** | **<0.0001** |
|  | **IFN-g/IL-2 T-cells** | **0.196** | **0.0002** | **0.2128** | **<0.0001** |

**Supplementary Table 4.** Differences between SARS-CoV2-specific immune responses between different immunosuppressive treatments at different time points.

|  |  | **CNI+MMF** | **MMF** | **CNI** | **CNI/mTOR-i** | **IC** | **P Value** |
| --- | --- | --- | --- | --- | --- | --- | --- |
| **IgG Spike titers**  **(BAU/mL)** | **T0** | 0 [0-0] | 0 [0-0] | 0 [0-0] | 0 [0-0] | 0 [0-0] | p=0.060 |
|  | **T1** | 0 [0-0] | 0 [0-0] | 111.28 [9.17-389.35] | 54.47 [1.31-300.95] | 221.78 [130.39-446.70] | **p<0.001** |
|  | **T2** | 16.82 [0-122.01] | 14.30 [0-236] | 1200 [41.34-2080] | 1551.16 [87.30-2080] | 01350 [815.50-1975] | **p<0.001** |
|  | **T3** | 17 [0-104.99] | 0 [0-68.59] | 299 [108.60-1250] | 818.01 [19.99-2060.01] | 451 [236-572.49] | **p<0.001** |
|  | **T4** | 888.99 [18.46-2080] | 12.79 [0-2069.99] | 2080 [481.99-2080] | 2080 [1063-2080] | 2080 [2080-2080] | **p=0.003** |

|  |  | **CNI+MMF** | **MMF** | **CNI** | **CNI/mTOR-i** | **IC** | **P Value** |
| --- | --- | --- | --- | --- | --- | --- | --- |
| **RBD-sp mBc ratio (sp/total)** | **T0** | 0 [0-0] | 0 [0-0] | 0 [0-0] | 0 [0-0] | 0 [0-0] | p=0.552 |
|  | **T1** | 0 [0-0.001] | 0 [0-0] | 0.003 [0-0.016] | 0.001 [0-0.005] | 0.012 [0-0.025] | **p=0.001** |
|  | **T2** | 0 [0-0.003] | 0 [0-0.003] | 0.042 [0.005-0.082] | 0.024 [0.004-0.066] | 0.012 [0.002-0.054] | **p<0.001** |
|  | **T3** | 0 [0-0.012] | 0.001 [0-0.007] | 0.079 [0.010-0.120] | 0.047 [0.009-0.074] | 0.046 [0.023-0.105] | **p<0.001** |
|  | **T4** | 0.004 [0-0.116] | 0.030 [0-0.149] | 0.250 [0.015-0.393] | 0.124 [0.017-0.336] | 0.436 [0.195-1.280] | **p=0.003** |

|  |  | **CNI+MMF** | **MMF** | **CNI** | **CNI/mTOR-i** | **IC** | **P Value** |
| --- | --- | --- | --- | --- | --- | --- | --- |
| **IFN-γ T-cell (SFU/2·10^5^ PBMC)** | **T0** | 1.50 [0-8.50] | 0.50 [0-2] | 1 [0-5.25] | 7.50 [1-24] | 2.50 [1.25-9.50] | **p=0.010** |
|  | **T1** | 5 [1-11.63] | 1.75 [0-40.50] | 5 [1.50-13.75] | 9.25 [2-34.88] | 7 [1.75-9.25] | p=0.181 |
|  | **T2** | 6.50 [1.50-14] | 1.50 [0-14.50] | 9 [1.50-28.50] | 26.50 [9.63-51.88] | 11 [3.50-35.25] | **p=0.002** |
|  | **T3** | 6 [2-14.75] | 3.75 [0-24.13] | 8.50 [3-24.50] | 14.50 [8.88-41.38] | 21 [15.75-23.75] | **p=0.003** |
|  | **T4** | 13.50 [6-33.25] | 5 [1-19.50] | 9.50 [4.75-21.75] | 43 [10-69.25] | 36 [24.25-50.75] | p=0.061 |

|  |  | **CNI+MMF** | **MMF** | **CNI** | **CNI/mTOR-i** | **IC** | **P Value** |
| --- | --- | --- | --- | --- | --- | --- | --- |
| **IL-2 T-cell (SFU/2·10^5^ PBMC)** | **T0** | 0 [0-1] | 0 [0-1] | 0 [0-0.25] | 0 [0-0.50] | 0 [0-0.4] | p=0.869 |
|  | **T1** | 2 [0-5.50] | 0.50 [0-13] | 13.50 [1.25-29] | 10.50 [2.88-43.50] | 8.50 [5.50-17] | **p=0.001** |
|  | **T2** | 11.50 [2.50-30] | 4.50 [1.50-14.50] | 34.50 [5.25-70.50] | 36.50 [5.13-106.13] | 11 [3.75-14.50] | **p=0.010** |
|  | **T3** | 11.50 [2.50-29] | 10.25 [0.38-31.50] | 26 [7.50-50] | 32.75 [10.38-64.38] | 23.50 [17-25.50] | p=0.051 |
|  | **T4** | 34 [9.75-89.25] | 6 [0-65.50] | 18 [4.25-47.50] | 93.50 [15.25-152] | 28 [23-49.75] | p=0.069 |

|  |  | **CNI+MMF** | **MMF** | **CNI** | **CNI/mTOR-i** | **IC** | **P Value** |
| --- | --- | --- | --- | --- | --- | --- | --- |
| **IFN-γ&IL-2 T-cell (SFU/2·10^5^ PBMC)** | **T0** | 0 [0-0.50] | 0 [0-1] | 0 [0-0] | 0 [0-0] | 0 [0-0] | p=0.307 |
|  | **T1** | 0 [0-0.50] | 0 [0-0.38] | 0.50 [0-2.25] | 0.75 [0-1.50] | 1 [0.75-2.50] | **p=0.003** |
|  | **T2** | 0.50 [0-2.50] | 0 [0-1.50] | 2 [0.50-7.50] | 2.25 [0.25-8.25] | 3.50 [0.50-5.75] | **p=0.005** |
|  | **T3** | 0.50 [0-2.25] | 0.50 [0-6] | 1.50 [0-4.50] | 2.25 [0.75-5.50] | 5 [3.50-7] | p=0.122 |
|  | **T4** | 3.50 [0.50-9] | 1 [0-8.50] | 2 [0-4.75] | 2 [0.50-13.75] | 6.50 [4.50-9] | p=0.314 |

|  |  | **CNI+MMF** | **MMF** | **CNI** | **CNI/mTOR-i** | **IC** | **P Value** |
| --- | --- | --- | --- | --- | --- | --- | --- |
| **IL-21 T-cell (SFU/2·10^5^ PBMC)** | **T0** | 0.50 [0-2.50] | 0.50 [0-1] | 0 [0-3] | 0 [0-1] | 0.50 [0-12.50] | p=0.798 |
|  | **T1** | 1.50 [0-9] | 0 [0-3.63] | 1 [0-27.75] | 14.50 [1.63-32.63] | 2 [0.75-16.25] | **p=0.018** |
|  | **T2** | 7 [1.50-20.50] | 1.50 [1-8] | 10.50 [1-55.75] | 27.25 [5.88-64.13] | 15.50 [3.25-30.75] | **p=0.045** |
|  | **T3** | 11 [2.50-33.75] | 5 [1-7] | 16.50 [1-39] | 19.75 [5.50-57.25] | 32 [9.50-76.25] | p=0.195 |
|  | **T4** | 36.50 [4-111.50] | 3 [1-14.50] | 21 [6.50-42.50] | 45.50 [10.50-97.50] | 25 [8.50-43.75] | p=0.132 |

|  |  | **CNI+MMF** | **MMF** | **CNI** | **CNI/mTOR-i** | **IC** | **P Value** |
| --- | --- | --- | --- | --- | --- | --- | --- |
| **IL-5 T-cell (SFU/2·10^5^ PBMC)** | **T0** | 0 [0-0.50] | 0 [0-0.50] | 0 [0-0.50] | 0 [0-1] | 0 [0-1] | p=0.981 |
|  | **T1** | 0.50 [0-1.63] | 0.25 [0-2.75] | 1 [0-3.75] | 3 [1-33.25] | 1.50 [0.75-4.25] | **p=0.003** |
|  | **T2** | 2.50 [0-13] | 0 [0-1] | 5.25 [1.88-11.13] | 24.25 [5.25-96] | 2 [0.50-23.50] | **p<0.001** |
|  | **T3** | 4 [0.50-11.50] | 1.50 [0-18.50] | 5 [0.50-13] | 23 [6-48.63] | 5 [1.25-7.50] | **p=0.003** |
|  | **T4** | 7 [1.13-31.25] | 1 [0-42] | 6 [0.25-25.25] | 21 [3.50-104.50] | 14.50 [8-39.75] | p=0.193 |

|  |  | **IgG Spike titers (BAU/mL)** | | | | **RBD-sp mBc (sp/total)** | | | | **IFN-γ T-cell (SFU/2·10^5^ PBMC)** | | | |
| --- | --- | --- | --- | --- | --- | --- | --- | --- | --- | --- | --- | --- | --- |
|  | **Time-point** | **MMF** | **CNI** | **CNI/mTOR-i** | **IC** | **MMF** | **CNI** | **CNI/mTOR-i** | **IC** | **MMF** | **CNI** | **CNI/mTOR-i** | **IC** |
| **CNI+MMF** | **T0** | **p=0.022** | **p=0.010** | p=0.258 | p=0.457 | p=1.000 | p=1.000 | p=0.494 | p=0.477 | p=0.181 | p=0.432 | **p=0.010** | p=0.090 |
|  | **T1** | p=0.201 | **p<0.001** | **p<0.001** | **p<0.001** | p=0.354 | **p<0.001** | **p=0.029** | **p<0.001** | p=0.733 | p=0.620 | **p=0.029** | p=0.287 |
|  | **T2** | p=0.976 | **p<0.001** | **p<0.001** | **p<0.001** | p=0.850 | **p<0.001** | **p<0.001** | **p<0.001** | p=0.367 | p=0.383 | **p<0.001** | **p=0.001** |
|  | **T3** | p=0.282 | **p<0.001** | **p<0.001** | **p<0.001** | p=0.980 | **p<0.001** | **p<0.001** | **p<0.001** | p=0.644 | p=0.143 | **p<0.001** | **p<0.001** |
|  | **T4** | p=0.175 | **p=0.011** | **p=0.018** | **p=0.001** | p=0.722 | **p=0.001** | **p=0.016** | **p<0.001** | p=0.141 | p=0.355 | p=0.054 | **p=0.006** |
| **MMF** | **T0** |  | p=0.947 | p=0.725 | p=0.680 |  | p=1.000 | p=0.998 | p=0.998 |  | p=0.363 | **p=0.008** | **p=0.028** |
|  | **T1** |  | **p<0.001** | **p=0.004** | **p<0.001** |  | **p=0.037** | p=0.060 | **p=0.011** |  | p=0.556 | p=0.355 | p=0.418 |
|  | **T2** |  | **p=0.019** | **p=0.008** | **p<0.001** |  | **p=0.007** | **p=0.005** | **p=0.001** |  | p=0.192 | **p=0.008** | **p=0.044** |
|  | **T3** |  | **p=0.001** | **p=0.004** | **p<0.001** |  | **p=0.004** | **p=0.003** | **p<0.001** |  | p=0.321 | p=0.077 | **p=0.031** |
|  | **T4** |  | **p=0.019** | **p=0.022** | **p=0.003** |  | p=0.063 | p=0.157 | **p=0.005** |  | p=0.348 | **p=0.030** | **p=0.006** |
| **CNI** | **T0** |  |  | p=0.400 | p=0.210 |  |  | p=0.317 | p=0.800 |  |  | **p=0.004** | p=0.058 |
|  | **T1** |  |  | p=0.433 | **p=0.009** |  |  | p=0.224 | p=0.123 |  |  | p=0.125 | p=0.605 |
|  | **T2** |  |  | p=0.393 | p=0.192 |  |  | p=0.615 | p=0.633 |  |  | **p=0.022** | p=0.064 |
|  | **T3** |  |  | p=0.620 | p=0.126 |  |  | p=0.382 | p=0.925 |  |  | **p=0.039** | **p=0.014** |
|  | **T4** |  |  | p=0.944 | p=0.575 |  |  | p=0.624 | p=0.062 |  |  | **p=0.042** | **p=0.002** |
| **CNI/mTOR-i** | **T0** |  |  |  | p=0.756 |  |  |  | p=0.157 |  |  |  | p=0.862 |
|  | **T1** |  |  |  | **p=0.005** |  |  |  | **p=0.014** |  |  |  | p=0.271 |
|  | **T2** |  |  |  | p=0.831 |  |  |  | p=0.303 |  |  |  | p=0.267 |
|  | **T3** |  |  |  | p=0.991 |  |  |  | p=0.385 |  |  |  | p=0.524 |
|  | **T4** |  |  |  | p=0.654 |  |  |  | **p=0.029** |  |  |  | p=0.920 |

|  |  | **IL-2 T-cell (SFU/2·10^5^ PBMC)** | | | | **IFN-γ&IL-2 T-cell (SFU/2·10^5^ PBMC)** | | | | **IL-21 T-cell (SFU/2·10^5^ PBMC)** | | | |
| --- | --- | --- | --- | --- | --- | --- | --- | --- | --- | --- | --- | --- | --- |
|  | **Time-point** | **MMF** | **CNI** | **CNI/mTOR-i** | **IC** | **MMF** | **CNI** | **CNI/mTOR-i** | **IC** | **MMF** | **CNI** | **CNI/mTOR-i** | **IC** |
| **CNI+MMF** | **T0** | p=0.771 | p=0.414 | p=0.834 | p=0.409 | p=0.467 | p=0.122 | p=0.396 | p=0.075 | p=0.815 | p=0.631 | p=0.399 | p=0.493 |
|  | **T1** | p=0.705 | **p=0.002** | **p=0.002** | **p<0.001** | p=0.469 | **p=0.001** | **p=0.018** | **p<0.001** | p=0.159 | p=0.505 | **p=0.005** | p=0.148 |
|  | **T2** | p=0.371 | **p=0.012** | **p=0.017** | p=0.064 | p=0.196 | **p=0.008** | **p=0.020** | **p<0.001** | p=0.136 | p=0.313 | **p=0.026** | p=0.839 |
|  | **T3** | p=0.657 | p=0.060 | **p=0.025** | **p=0.008** | p=0.905 | p=0.132 | **p<0.001** | **p<0.001** | p=0.076 | p=0.977 | **p=0.026** | p=0.520 |
|  | **T4** | p=0.111 | p=0.140 | p=0.166 | p=0.523 | p=0.496 | p=0.132 | p=0.479 | p=0.182 | **p=0.044** | p=0.379 | p=0.166 | p=0.083 |
| **MMF** | **T0** |  | p=0.888 | p=0.887 | p=0.410 |  | p=0.154 | p=0.282 | p=0.061 |  | p=0.558 | p=0.408 | p=0.948 |
|  | **T1** |  | p=0.085 | p=0.112 | **p=0.034** |  | p=0.068 | p=0.118 | **p=0.037** |  | p=0.120 | **p=0.009** | **p=0.046** |
|  | **T2** |  | p=0.075 | p=0.053 | p=0.086 |  | **p=0.023** | **p=0.014** | **p=0.008** |  | p=0.110 | **p=0.029** | **p=0.048** |
|  | **T3** |  | p=0.209 | p=0.117 | p=0.161 |  | p=0.759 | p=0.444 | p=0.070 |  | p=0.168 | **p=0.027** | p=0.064 |
|  | **T4** |  | p=0.410 | **p=0.035** | p=0.404 |  | p=0.787 | p=0.248 | p=0.164 |  | p=0.059 | **p=0.029** | p=0.382 |
| **CNI** | **T0** |  |  | p=0.626 | p=0.230 |  |  | p=0.676 | p=0.278 |  |  | p=0.689 | p=0.341 |
|  | **T1** |  |  | p=0.626 | p=0.526 |  |  | p=0.789 | p=0.838 |  |  | p=0.157 | p=0.697 |
|  | **T2** |  |  | p=0.557 | p=0.298 |  |  | p=0.831 | p=0.538 |  |  | p=0.324 | p=0.532 |
|  | **T3** |  |  | p=0.513 | p=0.858 |  |  | p=0.373 | **p=0.013** |  |  | p=0.355 | p=0.468 |
|  | **T4** |  |  | p=0.063 | p=0.280 |  |  | p=0.132 | **p=0.005** |  |  | p=0.235 | p=0.287 |
| **CNI/mTOR-i** | **T0** |  |  |  | p=0.357 |  |  |  | p=0.187 |  |  |  | p=0.386 |
|  | **T1** |  |  |  | p=0.984 |  |  |  | p=0.662 |  |  |  | **p=0.039** |
|  | **T2** |  |  |  | p=0.297 |  |  |  | p=0.649 |  |  |  | **p=0.045** |
|  | **T3** |  |  |  | p=0.586 |  |  |  | p=0.227 |  |  |  | p=0.660 |
|  | **T4** |  |  |  | p=0.248 |  |  |  | p=0.817 |  |  |  | p=0.069 |

|  |  | **IL-5 T-cell (SFU/2·10^5^ PBMC)** | | | |
| --- | --- | --- | --- | --- | --- |
|  | **Time-point** | **MMF** | **CNI** | **CNI/mTOR-i** | **IC** |
| **CNI+MMF** | **T0** | p=0.860 | p=0.988 | p=0.707 | p=0.756 |
|  | **T1** | p=0.889 | p=0.227 | **p<0.001** | **p=0.001** |
|  | **T2** | p=0.126 | p=0.171 | **p<0.001** | p=0.054 |
|  | **T3** | p=0.698 | p=1.000 | **p<0.001** | p=0.779 |
|  | **T4** | p=0.504 | p=0.563 | p=0.071 | p=0.427 |
| **MMF** | **T0** |  | p=0.885 | p=0.921 | p=0.708 |
|  | **T1** |  | p=0.467 | p=0.065 | p=0.095 |
|  | **T2** |  | **p=0.024** | **p=0.002** | **p=0.006** |
|  | **T3** |  | p=0.776 | **p=0.025** | p=0.609 |
|  | **T4** |  | p=0.810 | p=0.113 | p=0.811 |
| **CNI** | **T0** |  |  | p=0.724 | p=0.753 |
|  | **T1** |  |  | **p=0.021** | p=0.071 |
|  | **T2** |  |  | **p=0.004** | p=0.788 |
|  | **T3** |  |  | **p=0.001** | p=0.882 |
|  | **T4** |  |  | p=0.055 | p=0.915 |
| **CNI/mTOR-i** | **T0** |  |  |  | p=0.633 |
|  | **T1** |  |  |  | p=0.261 |
|  | **T2** |  |  |  | **p=0.005** |
|  | **T3** |  |  |  | **p=0.001** |
|  | **T4** |  |  |  | **p=0.029** |

**Supplementary Table 5.** Differences between SARS-CoV2-specific global responses (Humoral memory + T-cell memory) between different immunosuppressive treatments at different time points.

|  |  | **Global response** | | | |
| --- | --- | --- | --- | --- | --- |
|  | **Time-point** | **MMF** | **CNI** | **CNI/mTOR-i** | **IC** |
| **CNI+MMF** | **T0** | p=0.112 | p=0.692 | **p=0.005** | p=0.096 |
|  | **T1** | p=0.734 | **p<0.001** | **p=0.005** | **p<0.001** |
|  | **T2** | **p=0.017** | **p<0.001** | **p<0.001** | **p<0.001** |
|  | **T3** | p=0.125 | **p=0.001** | **p=0.037** | **p<0.001** |
|  | **T4** | p=0.177 | **p=0.047** | p=0.306 | **p=0.001** |
| **MMF** | **T0** |  | p=0.153 | **p=0.006** | p=0.323 |
|  | **T1** |  | p=0.189 | **p=0.047** | **p<0.001** |
|  | **T2** |  | **p=0.017** | **p=0.009** | **p<0.001** |
|  | **T3** |  | p=0.104 | **p=0.036** | **p<0.001** |
|  | **T4** |  | p=0.440 | p=0.188 | **p=0.001** |
| **CNI** | **T0** |  |  | **p=0.007** | p=0.246 |
|  | **T1** |  |  | **p=0.023** | **p=0.004** |
|  | **T2** |  |  | p=0.256 | p=0.066 |
|  | **T3** |  |  | p=0.318 | p=0.153 |
|  | **T4** |  |  | p=0.490 | **p=0.046** |
| **CNI/mTOR-i** | **T0** |  |  |  | **p<0.001** |
|  | **T1** |  |  |  | p=0.064 |
|  | **T2** |  |  |  | p=0.128 |
|  | **T3** |  |  |  | p=0.080 |
|  | **T4** |  |  |  | p=0.055 |

**Supplementary Table 6.** Statistical differences observed between groups in the Heatmap generated by hierarchical clustering of different SARS-CoV-2–specific memory immune response for solid organ transplant (SOT) and immunocompetent (IC) patients, according to the development of SARS-CoV-2-specific NAb. Immune responses used for clustering were differentially expressed (fold change >2, false discovery rate (fdr), p < 0.05). IFN, interferon; IL, interleukin; mBc, memory B cells.

|  | **Fold change** | **Log2r** | **P-value** | **False discovery rate** |
| --- | --- | --- | --- | --- |
| **RBD-sp mBc** | 27.32 | 4.77 | 0.0000 | 0.0000 |
| **IFN-γ T-cells** | 3.10 | 1.64 | 0.0000 | 0.0000 |
| **IL-2 T-cells** | 5.33 | 2.41 | 0.0000 | 0.0000 |
| **IFN-γ/IL-2 T-cells** | 6.85 | 2.78 | 0.0001 | 0.0001 |
| **IL-21 T-cells** | 1.21 | 0.27 | 0.0487 | 0.0487 |
| **IL-5 T-cells** | 4.87 | 2.28 | 0.0002 | 0.0002 |
| **mBc/IL-2 T-cells** | 2.12 | 1.08 | 0.0000 | 0.0000 |

|  | **SEVERE** | **MILD** | **ASYMPTOMATIC** | **NON-INFECTED** |
| --- | --- | --- | --- | --- |
| **SARS-CoV-2 specific IgG titers** | 15.50 [0-2080] | 1929.99 [247.42-2080] | 2080 [128.51-2080] | 2080 [95.95-2080] |
| **RBD specific mBc** | 0 [0-0.001] | 0.07 [0.01-0.23] | 0.23 [0.05-0.44] | 0.08 [0-0.32] |
| **IFN-γ T-cell (SFU/2·10^5^ PBMC)** | 3.75 [1.25-18.38] | 14.50 [8.75-29.63] | 21.25 [7.13-39.13] | 16 [6.25-42.50] |
| **IL-2 T-cell (SFU/2·10^5^ PBMC)** | 7.25 [0.50-51] | 42.25 [15.75-88.38] | 58 [24.13-90.38] | 29.50 [9.75-78.50] |
| **IFN-γ/IL-2 T-cell (SFU/2·10^5^ PBMC)** | 0 [0-2.88] | 4 [1.50-7.13] | 5 [1.63-10] | 2 [0.50-8.25] |
| **IL-21 T-cell (SFU/2·10^5^ PBMC)** | 12.50 [0-104.38] | 18.75 [4.50-109.63] | 13.50 [7-63] | 34 [3.25-88.50] |
| **IL-5 T-cell (SFU/2·10^5^ PBMC)** | 1.75 [0-15.75] | 10 [1.75-17.38] | 14 [4-61] | 6 [0.75-29.75] |

**Supplementary Table 7.** Differences between immune cell compartments and distinct clinical COVID-19 breakthrough infections.

|  | **IgG Spike titers (BAU/mL)** | | | | **RBD-sp mBc (sp/total)** | | | | **IFN-γ T-cell (SFU/2·10^5^ PBMC)** | | | |
| --- | --- | --- | --- | --- | --- | --- | --- | --- | --- | --- | --- | --- |
|  | **SEV** | **MILD** | **ASYMP** | **NO-INF** | **SEV** | **MILD** | **ASYMP** | **NO-INF** | **SEV** | **MILD** | **ASYMP** | **NO-INF** |
| **SEVERE** |  | **p=0.036** | **p=0.025** | **p=0.028** |  | **p<0.001** | **p<0.001** | **p=0.001** |  | **p=0.044** | **p=0.035** | **p=0.033** |
| **MILD** |  |  | p=0.665 | p=0.953 |  |  | p=0.044 | p=0.996 |  |  | p=0.641 | p=0.979 |
| **ASYMP** |  |  |  | p=0.583 |  |  |  | **p=0.043** |  |  |  | p=0.681 |
|  | **IL-2 T-cell (SFU/2·10^5^ PBMC)** | | | | **IFN-γ/IL-2 T-cell (SFU/2·10^5^ PBMC)** | | | | **IL-21 T-cell (SFU/2·10^5^ PBMC)** | | | |
|  | **SEV** | **MILD** | **ASYMP** | **NO-INF** | **SEV** | **MILD** | **ASYMP** | **NO-INF** | **SEV** | **MILD** | **ASYMP** | **NO-INF** |
| **SEVERE** |  | p=0.066 | **p=0.045** | p=0.108 |  | **p=0.013** | **p=0.016** | p=0.057 |  | p=0.470 | p=0.569 | p=0.336 |
| **MILD** |  |  | p=0.623 | p=0.353 |  |  | p=0.614 | p=0.332 |  |  | p=0.676 | p=0.893 |
| **ASYMP** |  |  |  | p=0.147 |  |  |  | p=0.176 |  |  |  | p=0.572 |
|  | **IL-5 T-cell (SFU/2·10^5^ PBMC)** | | | |  |  |  |  |  |  |  |  |
|  | **SEV** | **MILD** | **ASYMP** | **NO-INF** |  |  |  |  |  |  |  |  |
| **SEVERE** |  | p=0.124 | **p=0.024** | p=0.183 |  |  |  |  |  |  |  |  |
| **MILD** |  |  | p=0.219 | p=0.569 |  |  |  |  |  |  |  |  |
| **ASYMP** |  |  |  | p=0.089 |  |  |  |  |  |  |  |  |

**Supplementary Table 8.** Demographic, clinical variables and SARS-CoV-2-specific memory immune responses after vaccination (T4) predicting severe COVID-19 BTI.

| **Variables** | **Univariate Analysis** | | | **Multivariate Analysis** | | |
| --- | --- | --- | --- | --- | --- | --- |
|  | **HR** | **95% CI** | **p-value** | **OR** | **95% CI** | **p-value** |
| **Age** | **1.038** | **0.999-1.077** | **0.053** | 1.050 | 0.983-1.050 | 0.148 |
| **Time after Transplant** | 0.995 | 0.986-1.003 | 0.191 | NA | NA | NA |
| **Steroids use** | 1.149 | 0.859-1.537 | 0.349 | NA | NA | NA |
| **CNI Trough** | 1.185 | 0.913-1.538 | 0.201 | NA | NA | NA |
| **rATG induction** | 0.418 | 0.106-1.649 | 0.213 | NA | NA | NA |
| **SARS-CoV-2 Spike IgG** | **0.183** | **0.057-0.588** | **0.004** | 1.245 | 0.230-6.745 | 0.799 |
| **SARS-CoV-2 specific mBc** | **0.074** | **0.019-0.291** | **0.001** | **0.097** | **0.018-0.533** | **0.007** |
| **SARS-CoV-2 specific IFN-y T-cells** | **0.174** | **0.053-0.569** | **0.004** | 0.393 | 0.082-1.891 | 0.244 |
| **SARS-CoV-2 specific IL-2 T-cells** | **0.190** | **0.059-0.618** | **0.006** | 0.599 | 0.123-2.908 | 0.525 |

**Abbreviations: CNI: calcineurin inhibitors; rATG: rabbit anti-Thymocyte globulin; IgG: immunoglobulin G; mBc: memory B cells**

**Supplementary Figure 1.** Flow-chart of the randomized controlled trial (Tor-vax study).

**
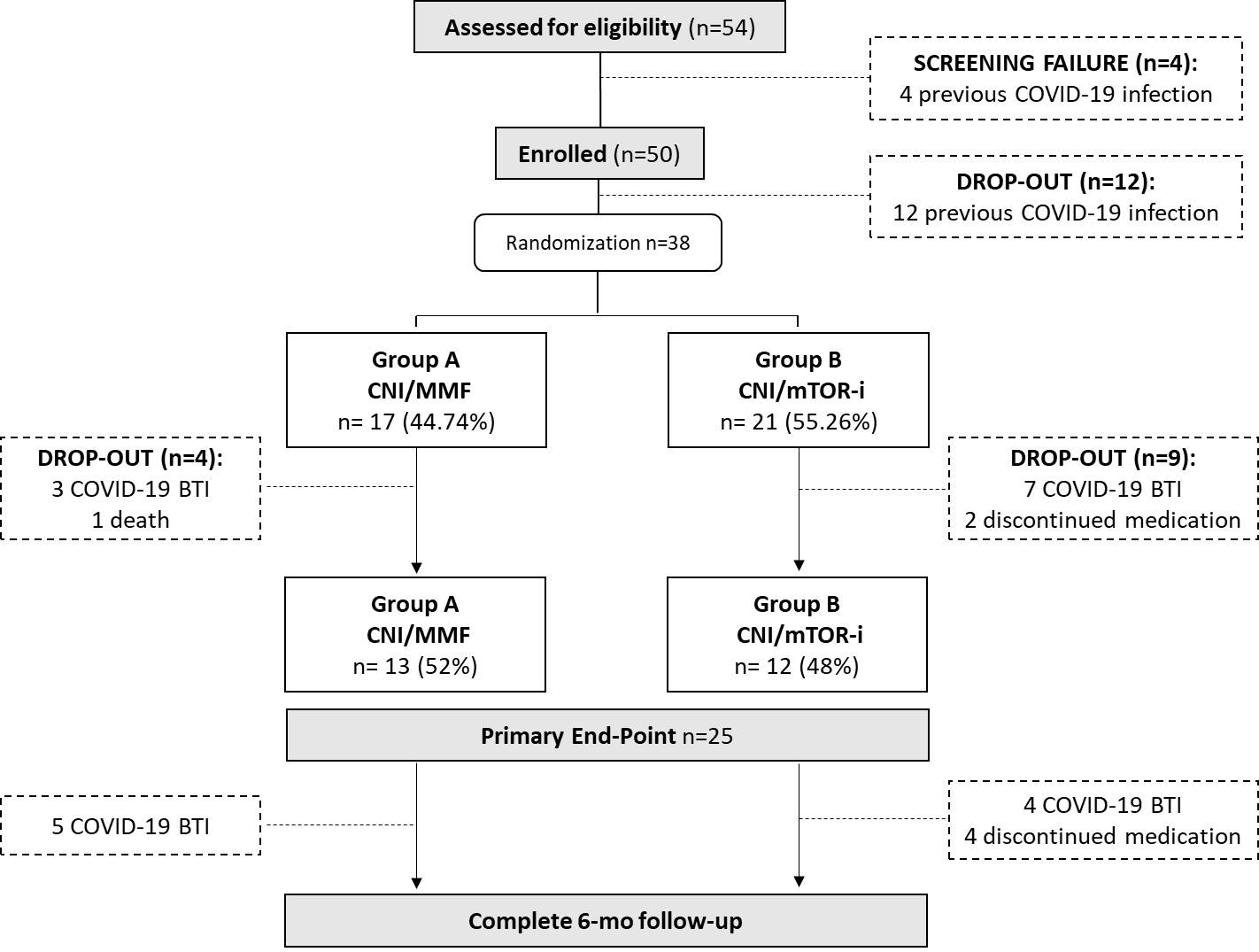
**

**

Supplementary Figure 2.** SARS-CoV-2-specific IgG antibody titers and neutralizing activity. *p<0.005.

**Supplementary Figure 3.** Spearman correlations between IgG levels in serum (BAU/mL) and frequencies of memory B cells (ratio between specific mBc/4.5·10^4^ PBMC). (**A**) 28 days after 1^st^ dose: T1. (**B**) 2 months after 2^nd^ dose: T2. (**C**) 5 months after the 2^nd^ dose: T3. (**D**) 1 month after the 3^rd^ dose: T4 (only for SOT patients).

**
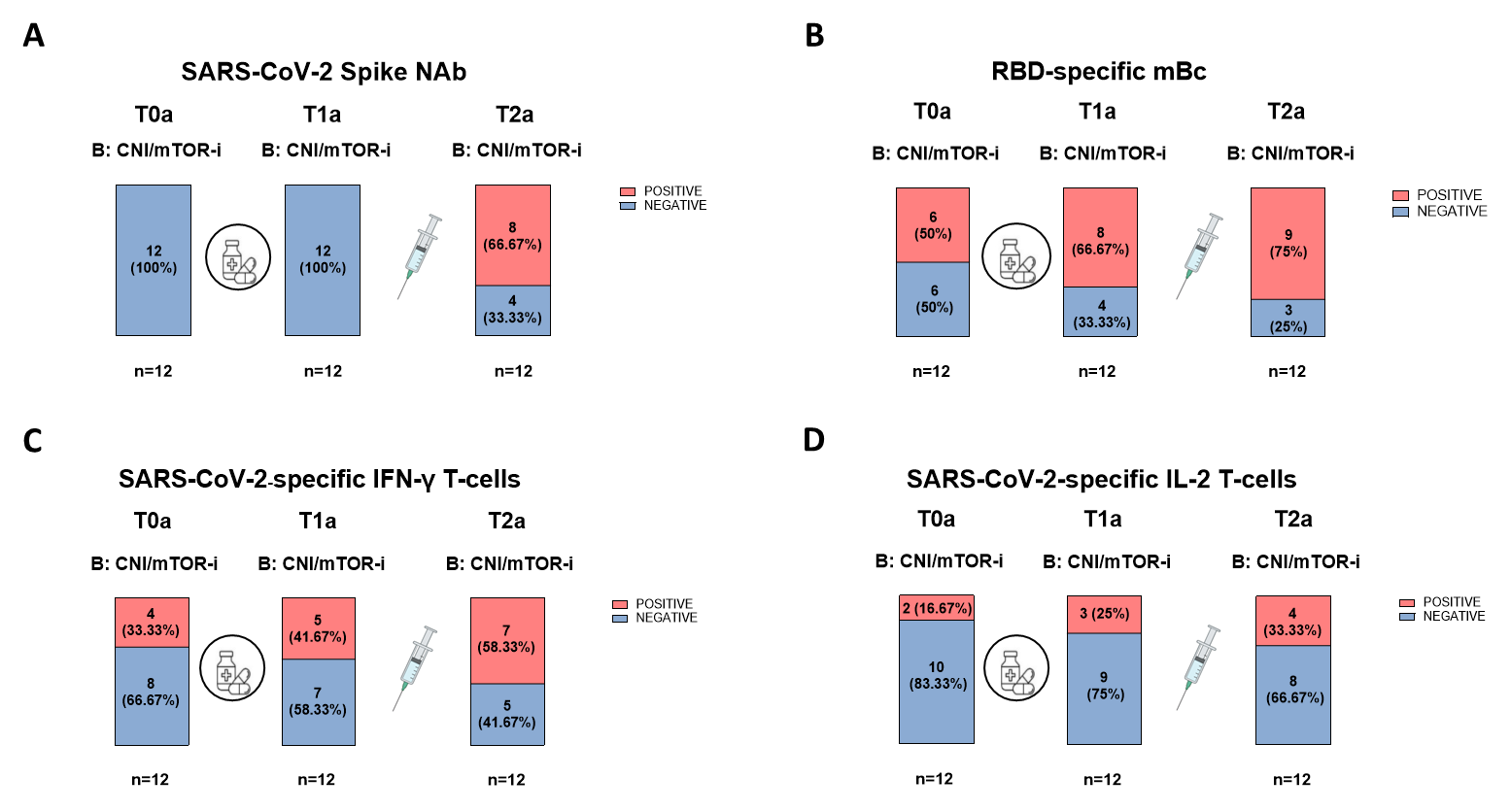
Supplementary Figure 4.** Results of the randomized controlled trial. Presence of neutralizing antibodies (**A**), RBD-specific memory B cells (**B**), SARS-CoV-2-specific IFN-γ T-cells (**C**) and SARS-CoV-2-specific IL-2 T-cells (**D**) in patients from group B at the time of randomization, one month after randomization and one month after the 4^th^ vaccine dose.

**Supplementary Figure 5.** Spearman correlations between IgG levels in serum (BAU/mL) and frequencies of memory B cells (ratio between specific mBc/4.5·10^4^ PBMC). (**A**) 1 month after 4^th^ dose: T2a. (**B**) Frequencies of RBD-specific mBc prior to booster vaccination (T0a) and levels of Spike IgG in serum at T2a, after the 4^th^ booster vaccine.

**Supplementary methods**

**Randomized clinical trial (Tor-Vax trial)**

The flow-chart of the study is shown in **Figures 1** and **Supplementary Figure 2**.

Clinical variables analysed:

Renal function evolution, incidence of acute rejection, development of de novo donor-specific antibodies (DSA) and drug related adverse effect during follow-up were recorded in each center.

*Immunosupression*

Chronic maintenance immunosuppression was homogenous in all included patients previous to randomization and was based on CNI (tacrolimus), MMF/MPS, with or without corticosteroids. After randomization, patients in Group A maintained the initial immunosuppressive treatment (Tacrolimus trough levels 5-8 ng/mL; MMF/MPA dose 500 or 360 mg/12h, respectively) and patients in Group B were converted from MMF/MPS to the mTOR-inhibitor sirolimus (Rapamune®, Pfizer) (initial dose 1 mg daily, to achieve trough levels 3-5 ng/mL), maintaining tacrolimus (trough levels 4-6 ng/mL), with or without corticosteroids.

*SARS-CoV-2 vaccination*

All included patients received a fourth dose of BNT162b2 mRNA SARS-CoV-2 intramuscular vaccine (Pfizer-BioNTech) intramuscular in the upper arm between May and July 2022.

**Assessment of SARS-CoV-2-specific Adaptive Immune Memory**

**A. Assessment of SARS-CoV-2-specific Humoral Memory**

**A.1 SARS-CoV-2-specific Serological Memory**

SARS-CoV-2-specific serum IgG antibodies.

Briefly, serological response to SARS-CoV-2 was determined by the detection of specific antibodies against both nucleocapsid and spike SARS-CoV-2 antigens. Two commercial chemiluminescence immunoassays (CLIA) were used, according to the manufacture instructions:

1. Elecsys® Anti-SARS-CoV-2 (Roche Diagnostics, Mannheim, Germany) performed on the Cobas 8800 system (Roche Diagnostics, Switzerland) for the determination of total antibodies (including IgG, IgM and IgA) against nucleocapsid SARS-CoV-2 proteins (cut-off ≥ 1.0 index). Human serum reactive (ACOV2 Cal2) and non-reactive (ACOV1 Cal1) for SARS-CoV-2 antibodies were used as negative and positive calibrators (https://www.fda.gov/media/137605/download).
2. LIAISON® SARS-CoV-2 TrimericS IgG (DiaSorin, Stillwater, MN, USA) performed on the LIAISON® XL Analyzer (DiaSorin, Italy) for the determination of IgG antibodies spike glycoprotein of SARS-CoV-2 (cut-off ≥ 13.0 AU/mL; measures up to 800 AU/mL). Human serum reactive and non-reactive for SARS-CoV-2 IgG antibodies 0.2% ProClin 300 were used as internal negative and positive controls, as provided by the manufacturer (https://www.fda.gov/media/149059/download). Results were provided using BAU/mL units according to manufacture instructions.

SARS-CoV-2-specific Neutralizing Antibodies

Neutralizing antibodies were evaluated against SARS-CoV-2 Wuhan (D614G variant) and Omicron (BA.5 variant). Briefly, serum and supernatants resulting from mBc culture (see section A.2) from transplanted patients were used to neutralize a Pseudotyped VSV-SARS-CoV-2 Spike expressing a luciferase (VSV-ΔG-Luc-S). Pseudyotyped viral stocks of VSV-ΔG-Luc-S were generated following the protocol described by Whitt with the modifications previously described [1].

Plasma samples were heat-inactivated at 56ºC for 30 minutes. VeroE6-TMPRSS2 were obtained from the National Institute for Biological Standards and Control (NIBSC). Cells were maintained in Dulbecco’s Modified Eagle Medium (High Glucose) with 2 mM L-glutamine (Lonza, Basel, Switzerland) and 100X penicillin/streptomycin (Lonza, Basel, Switzerland) supplemented with 10% of Fetal Bovine Serum (FBS, Corning, Corning, US). After, plasma and supernatant samples were diluted four-fold towards a concentration of 1/32 of the initial sample with culture medium supplemented by 2% of FBS, 10 mM HEPES (1M, Hyclone, Cytiva) and 1 mg/mL of G-418. Diluted plasma samples were then incubated with pseudotyped virus (VSV-ΔG-Luc-S) with titers of approximately 1x10^6^-5x10^5^ RLUs/mL of luciferase activity in a 96 well-plate flat bottom for 1h at 37ºC, 5% CO_2_ to promote virus neutralization. VeroE6-TMPRSS2 cells were added at a density of 30.000 cells/well and incubated with neutralized virus at 37ºC overnight. The next day, cells were incubated with Britelite plus reagent (Britelite plus kit; PerkinElmer, Waltham, Massachusetts, US) and then transferred to an opaque black plaque. Luminescence was immediately recorded by a luminescence plate reader (LUMIstar Omega).

Both a negative (uninfected cells) and a positive (100% infection) controls had been included in each plate. Moreover, a negative serum from SARS-CoV-2 antibodies was also added. Each experiment was performed in duplicate.

Viral neutralization was calculated as the reciprocal plasma dilution (ID50) resulting in a 50% reduction in relative light units. If no neutralization was observed, an arbitrary titer value of <16 (half of the limit of detection [LOD]) was reported.

**A.2 SARS-CoV-2-specific IgG-producing Memory B cells**

Briefly, to differentiate circulating mBcs to antibody-secreting cells (ASCs), peripheral blood mononuclear cells were cultured (1.5×10^6^ cells per mL at 37 °C in 5% CO2) for 6 days in Iscove Modified Dulbecco Media (IMDM) enriched medium, 500 ng/mL Human CD40/TNFRSF5 Antibody (Bio-Techne R&D Systems, S.L.U., USA), 600 IU/mL human interleukin-2 (Sigma Aldrich, USA), 100 ng/ml human interleukin-21 (Peprotech, UK), 25 ng/mL human interleukin-10 (Peprotech, UK), 2.5 ug/mL CpG-B DNA (ODN 2006) (HycultBiotech, The Netherlands) and 10 ul/mL ITS Liquid Media Supplement (Sigma Aldrich, USA), as previously described by our group [4].

After 6-day stimulation, 4.5×10^5^ stimulated cells were seeded in each well to assess SARS-CoV-2 specific IgG spots, whereas 4.5×10^4^ and 4.5×10^3^ stimulated cells were seeded to assess the polyclonal IgG spot detection.

For the detection of specific SARS-CoV-2 mBcs, we used RBD-WASP (recombinant SARS-CoV-2 Receptor Binding Domain (RBD) of the Spike protein (aa 319-541) with a C-terminal WASP peptide tag [PDYRPYDWASPDYRD]) at 1:20 dilution; followed by anti-WASP-HRP (horseradish peroxidase) at 1:1000 dilution.

For the polyclonal IgG detection, mAbs MT78/145-Biotin (1 ug/mL) and Streptavidin-HRP (1:1000) were used, respectively (anti-human IgG MT78/145 is a mouse monoclonal antibody to IGHG1, IGHG2, IGHG4, and immunoglobulin heavy constant gamma 1 [G1m marker]; this biotinylated antibody interacts and binds to the streptavidin-HRP complex to ultimately generate a detectable signal).

Next, 100 uL of ready-to-use TMB (3,3’, 5,5’-tetramethylbenzidine) solution was used as substrate for HRP, in order to develop the reaction until distinct spots emerge. After the plate was dried, spots were count in the Fluorspot Reader version 8 (AID® Gmbh, Strassberg, Germany). The ratio between RBD-specific mBcs over the total polyclonal IgG mBcs in each patient was used as a reliable approach to characterize the proportion or enhancement of a given RBD-specific IgG-antibody secreting cell (ASC) within the global IgG-ASC population. This approach method allows for qualitative and quantitative easy comparisons between sample stimulations [4, 5].

Any ELISPOT test with non-detectable RBD-specific spots were considered as negative when assessed in a qualitative manner. 5.24% of patients showed suboptimal proliferation results and were excluded from the analysis.

**B. SARS-CoV-2-reactive cytokine-producing Memory T cells**

Briefly, 2x10^5^ PBMCs (in 100 µl) were stimulated with SARS-CoV-2 Spike Glycoprotein (S) for 24 hours for 5 different cytokine-producing T-cell populations: effector (IFN-γ), proliferative (IL-2), central (IFN-γ/IL-2) Th1, proliferative (IL-21) Th2 and Th17, and stimulating (IL-5) Th2. After washing steps, the different cytokine fluorospots were detected using primary and secondary antibodies against each cytokine plus the addition of enhancer. The spots obtained were automatically counted with the Fluorospot Reader version 8 (AID® Gmbh, Strassberg, Germany).

The Spike Glycoprotein (S) overlapping peptide pool (P0DTC2 protein, S gene) contained 158 + 157 peptides of >70% purity and was reconstituted in DMSO and PBS and used at a final concentration of 2 µg/mL.

In each test, complete medium alone (20% Fetal Bovine Serum (FBS) and 80% RPMI solution) and Pokeweed (PWM) mitogen were used as negative and positive controls, respectively. The results were considered after subtracting to each well the responses obtained in the respective internal negative control well.

As external negative controls, we previously showed [6] that pre-pandemic unexposed individuals to SARS-CoV-2 did not respond to any of the used overlapping peptide pools of SARS-CoV-2 proteins (never exceeded 5 spots/2x10^5^ stimulated PBMCs).

**C. Analysis of Immune memory compartments**

SARS-CoV-2-specific immune memory responses were considered detectable for each immune compartment as follows: for T-cell immune memory responses, if any antigen-specific quantitative cytokine-producing T-cell frequency higher than 5 spots/2x10^5^ PBMC was observed after subtracting all unspecific SFU in each Fluorospot assay; for the B-cell compartment we considered when either there was presence of SARS-CoV-2 NAb and/or any detectable IgG-producing mBc frequency after subtracting all unspecific spots in each test.

**References**

1. [Grau-Expósito](https://doi.org/10.1371/journal.ppat.1010171) J, et al. Evaluation of SARS-CoV-2 entry, inflammation and new therapeutics in human lung tissue cells. *PLoS Pathog*. 2022;18(1):e1010171. [SM]
2. Ritz C, et al. Dose-Response Analysis Using R. *PLoS ONE*. 2015;10(12):e0146021. [SM]
3. R Core Team (2021). R: A language and environment for statistical computing. R Foundation for Statistical Computing, Vienna, Austria. URL https://www.R-project.org/. [SM]
4. Luque S, et al. A multicolour HLA-specific B-cell FluoroSpot assay to functionally track circulating HLA-specific memory B cells. *J Immunol Methods.* 2018;462:23-33. [SM]
5. Lúcia M, Luque S, Crespo E, et al. Preformed circulating HLA-specific memory B cells predict high risk of humoral refection in kidney transplantation. *Kidney Int.* 2015;88(4):874-887. [SM]
6. Favà A, Donadeu L, Sabé N, et al. SARS-CoV-2-specific serological and functional T cell immune responses during acute and early COVID-19 convalescence in solid organ transplant patients. *Am J Transplant*. 2021;21(8):2749-2761. [SM]
